# Differential spatial working memory–related functional network reconfiguration in young and older adults

**DOI:** 10.1162/netn_a_00358

**Published:** 2024-07-01

**Authors:** Wan Lin Yue, Kwun Kei Ng, Siwei Liu, Xing Qian, Joanna Su Xian Chong, Amelia Jialing Koh, Marcus Qin Wen Ong, Simon Kang Seng Ting, Adeline Su Lyn Ng, Nagaendran Kandiah, B. T. Thomas Yeo, Juan Helen Zhou

**Affiliations:** Centre for Sleep and Cognition & Centre for Translational Magnetic Resonance Research, Yong Loo Lin School of Medicine, National University of Singapore; Integrative Sciences and Engineering Programme, NUS Graduate School, National University of Singapore; National Neuroscience Institute, Singapore; Neuroscience and Behavioural Disorders Programme, Duke-NUS Medical School, Singapore; Martinos Center for Biomedical Imaging, Massachusetts General Hospital; Department of Electrical and Computer Engineering, N.1 Institute for Health and Memory Networks Program, National University of Singapore

**Keywords:** Aging, fMRI, Functional connectivity, Functional reconfiguration, Spatial working memory

## Abstract

Functional brain networks have preserved architectures in rest and task; nevertheless, previous work consistently demonstrated task-related brain functional reorganization. Efficient rest-to-task functional network reconfiguration is associated with better cognition in young adults. However, aging and cognitive load effects, as well as contributions of intra- and internetwork reconfiguration, remain unclear. We assessed age-related and load-dependent effects on global and network-specific functional reconfiguration between rest and a spatial working memory (SWM) task in young and older adults, then investigated associations between functional reconfiguration and SWM across loads and age groups. Overall, global and network-level functional reconfiguration between rest and task increased with age and load. Importantly, more efficient functional reconfiguration associated with better performance across age groups. However, older adults relied more on internetwork reconfiguration of higher cognitive and task-relevant networks. These reflect the consistent importance of efficient network updating despite recruitment of additional functional networks to offset reduction in neural resources and a change in brain functional topology in older adults. Our findings generalize the association between efficient functional reconfiguration and cognition to aging and demonstrate distinct brain functional reconfiguration patterns associated with SWM in aging, highlighting the importance of combining rest and task measures to study aging cognition.

## Author Summary

Brain networks identified by functional connectivity (FC) have preserved architectures from rest to task and across task demands. Higher similarity, implying more efficient network reconfiguration, was associated with better cognition and task performance in young adults. To examine how it may be influenced by aging, we compared whole-brain and network-level FC similarities between resting-state and spatial working memory fMRI in young and older adults. At whole-brain level and higher order cognitive networks, older adults evidenced less efficient network reconfiguration from rest to task than young adults. Importantly, more efficient reconfiguration was associated with better accuracy. This relationship relied more on internetwork connections in older adults. Despite reduced neural resources compared to young, maintaining efficient network updating still contributes to better cognition at older age.

## INTRODUCTION

The brain is active during both goal-oriented (task) and unconstrained task-free (rest) conditions (Shulman, Rothman, Behar, & Hyder, 2004). The brain functional communication and integration of information during both rest and task can be represented by functional connectivity (FC), which reflects the temporal synchrony of neuronal activity between different brain regions. FC during both rest and task have strong associations with performance across different cognitive domains (Andrews-Hanna et al., 2007; Finn et al., 2015; Greene, Gao, Scheinost, & Constable, 2018; Hampson, Driesen, Skudlarski, Gore, & Constable, 2006; Jiang et al., 2020; Rosenberg et al., 2020). Directly comparing rest FC and task FC revealed patterns of localized changes on a background of highly similar FC patterns between rest and task (Cole, Bassett, Power, Braver, & Petersen, 2014; Gratton, Laumann, Gordon, Adeyemo, & Petersen, 2016; Jurkiewicz, Crawley, & Mikulis, 2018; Krienen, Yeo, & Buckner, 2014; Lynch et al., 2018), leading to research on reconfiguration of functional brain networks from rest to task.

Previous work found that more efficient global reconfiguration of FC between rest and task (i.e., higher similarity between rest and task FC) was related to better performance for visual working memory, language, and reasoning tasks in young adults (Schultz & Cole, 2016; Zuo, Yang, Liu, Li, & Jiang, 2018), with the similarity metric explaining task performance beyond interindividual differences in rest or task FC alone (Schultz & Cole, 2016). This suggests that individuals with higher cognitive capacity possess resting FC already optimized for domain-general cognition. Furthermore, there is some evidence that the association may extend to higher order (e.g., executive control and default mode networks) and task-relevant (e.g., auditory and visual) networks, though previous work did not distinguish between reconfiguration at the intranetwork (between brain regions of the same network) and internetwork (between brain regions from different networks) levels. Associations of efficient network reconfiguration with cognition at the network level would be consistent with findings that intrinsic brain networks are implicated in functions involving specific cognitive domains (Corbetta & Shulman, 2002; Damoiseaux et al., 2006; Power et al., 2011; Yeo et al., 2011). More efficient reconfiguration of FC between working memory (WM) task loads was also associated with better task performance at both the whole brain and network level (Zuo et al., 2018). However, only one type of WM task (N-back) was used in earlier studies on functional reconfiguration (Schultz & Cole, 2016; Zuo et al., 2018), while different types of WM have been found to involve different neural circuits and cognitive processes (D’Esposito et al., 1998; Ren et al., 2019; Wager & Smith, 2003). For example, brain activation patterns for object WM and spatial WM tasks corresponded to different regions in the posterior cortex (Wager & Smith, 2003). Therefore, it remains unclear whether efficient functional network reconfiguration is also relevant to performance in other WM tasks.

Moreover, FC in both rest and task are also modulated by age, with reduced functional specialization (decreased intranetwork FC) and reduced brain functional network segregation (increased internetwork FC), often observed in older adults across rest and task, such as in the executive control, default mode, and salience networks (Andrews-Hanna et al., 2007; Betzel et al., 2014; Geerligs, Maurits, Renken, & Lorist, 2014; K. K. Ng, Lo, Lim, Chee, & Zhou, 2016; Spreng, Stevens, Viviano, & Schacter, 2016). These FC changes are also linked to age-related cognitive decline (Andrews-Hanna et al., 2007; Geerligs, Maurits, et al., 2014; K. K. Ng et al., 2016). Furthermore, changes in task FC across rest and task, and between task loads, are also modulated by age (Avelar-Pereira, Bäckman, Wåhlin, Nyberg, & Salami, 2017; Geerligs, Saliasi, Renken, Maurits, & Lorist, 2014; Salami, Pudas, & Nyberg, 2014; Sambataro et al., 2010). With task- and load-specific changes occurring on top of aging-related changes at resting state, functional reconfiguration between rest and task, as well as across task loads, may also be modulated by aging. The ability to maintain efficient functional reconfiguration could also be relevant to age-related cognitive decline. In the context of healthy aging, functional reconfiguration may be related to changes in specialization and segregation of network functions. Furthermore, age-related differences in functional reconfiguration across loads could also reflect changes in utilization of functional networks to adapt to higher cognitive loads, which relates to the Compensation-Related Utilization of Neural Circuits hypothesis (CRUNCH) (Reuter-Lorenz & Cappell, 2008).

However, since previous studies on functional reconfiguration focused on young adults (Schultz & Cole, 2016; Zuo et al., 2018), the effect of age on functional reconfiguration and its association to cognition remains unclear. Furthermore, distinguishing functional reconfiguration between rest and task from stepwise functional reconfiguration (i.e., between task loads) may allow us to interrogate the specific context in which aging impairs cognition.

To address those gaps, we first aimed to investigate age- and load-dependent differences in global- and network-level rest-task and stepwise functional reconfiguration between resting state and a spatial working memory (SWM) task with three memory loads. Next, we aimed to investigate the association between global functional brain reconfiguration and task performance in both young and older adults, then to investigate the network-specific contributions of intra- and internetwork reconfiguration to these associations. We hypothesized that more functional reconfiguration (i.e., lower FC similarity between rest and task) would be required as age and load increased, reflecting increased difficulty in performing the task in aging and at higher task loads. We further hypothesized that higher FC similarity of both global- and network-level functional organization would be associated with better task performance regardless of age group. We expected that higher order cognitive networks and spatial WM task-relevant networks would have stronger contributions to these associations.

## METHODS

### Participants

To investigate the age effect on rest-task functional reconfiguration, we used data collected from healthy young adults and healthy older adults across two datasets and selected a total of 109 young adults and 34 healthy older adults for analyses based on the following procedures (Table 1). The young adults were from a dataset described in Liu et al. (2018), while older adults were healthy controls from a dataset described in previous work (A. S. L. Ng et al., 2021; Tan et al., 2017; Vipin et al., 2018; Yatawara, Lim, Chander, Zhou, & Kandiah, 2016). All participants performed the same SWM task which had three task loads (SWM load), allowing us to investigate the load effect on functional reconfiguration. The young adults were recruited from National University of Singapore and the community, while older adults were recruited from the National Neuroscience Institute in Singapore. These studies were approved by the National University of Singapore Institutional Review Board and the SingHealth Institutional Review Board respectively. Written informed consent was obtained from all participants.

**Table 1.** Participant demographics and motion parameters

	***N***	**Age (years)**	**Sex (M/F)**	**Handedness (R/L/A)**	**MMSE**	**MoCA**	**Mean relative motion (mm)**		**Mean absolute motion (mm)**	
							Rest	Task	Rest	Task
Young adults	109	24.8 (4.2)	52/57	107/2/0	–	–	0.05 (0.02)	0.05 (0.02)	0.25 (0.18)	0.18 (0.12)
Older adults	34	65.1 (6.6)	18/16	31/1/2	28.8 (1.3)	28.5 (1.2)	0.07 (0.03)	0.07 (0.04)	0.32 (0.20)	0.27 (0.23)

*Note*. Age, MMSE scores, MoCA scores, and motion parameters are presented as mean (standard deviation). M/F = male/female; R/L/A = right/left/ambidextrous; MMSE = Mini Mental State Examination; MoCA = Montreal Cognitive Assessment.

To include only healthy participants, older adults were required to meet these criteria for inclusion in this study: (1) Mini Mental State Examination (MMSE) score ≥ 26 and Montreal Cognitive Assessment (MoCA) score ≥ 26; (2) clinical dementia rating (CDR) = 0; (3) no history of significant vascular events; (4) no history of malignant neoplasia; (5) no history of organ failure (heart, lung, liver, or kidney); (6) no thyroid disease (active or inadequately treated); (7) no active neurological or psychiatric conditions; and (8) no history of head trauma with loss of consciousness, resulting in 187 older adult participants being excluded. For young adults, the requirements for inclusion in this study were the following: (1) age between 18 and 35; (2) have no history of psychiatric or neurologic disorders; and (3) no long-term medications of antipsychotics, anxiolytics, antidepressants, resulting in 21 young adult participants being excluded. Medical history for both datasets were based on self-reports. All participants were also required to have performance above chance level (accuracy > 0.5) for the easy task load in the SWM-load task, as well as functional magnetic resonance imaging (fMRI) data for resting state and SWM-load task that met quality control criteria (see section Image Preprocessing for details), with another 61 older adult participants and 21 young adult participants being excluded. These criteria resulted in data from 109 young adults and 34 healthy older adults included in the final analyses (Table 1).

### Spatial Working Memory Task

**Figure 1.**
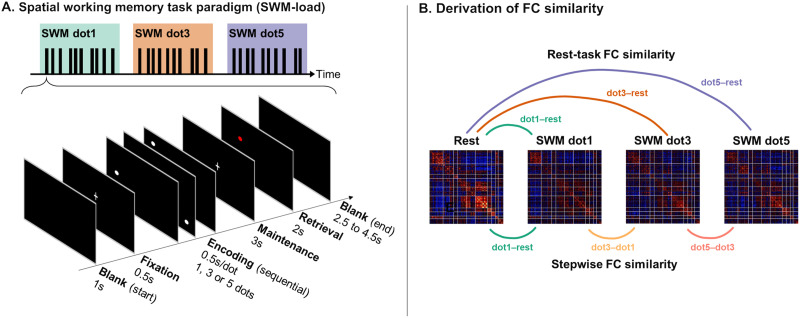
Spatial working memory (SWM) task paradigm and functional connectivity (FC) similarity. (A) Structure of a single run (top) and of a single trial (bottom) in the SWM-load task. Each task run consisted of one block of each task load, with one block containing 10 trials. (B) Illustration for derivation of rest-task FC similarity (top) and stepwise FC similarity (bottom). Rest-task FC similarity was calculated between rest FC and task FC at each task load. Stepwise FC similarity was calculated between FC from a higher task load to the next (lower) load.

We used a variant of SWM task based on the Sternberg delayed-match-to-sample task paradigm (Manoach, Greve, Lindgren, & Dale, 2003; Sternberg, 1966) to investigate age-related differences in functional network reconfiguration. The SWM-load task (Figure 1A) consisted of three memory loads of one, three, or five dots, respectively. Each trial started with a blank (1 s), followed by fixation (0.5 s). Dots were presented at random positions (in a 5 × 5 grid) during encoding (0.5 s per dot), with the number of dots differing according to task load. This was followed by a maintenance period (3 s), after which a probe dot was presented for retrieval (2 s). Participants were then required to respond via button press whether the probe dot was in the same position as any of the dots shown during encoding. A blank screen was presented between trials with randomly jittered intervals (2.5 s to 4.5 s). Each SWM-load task run comprised three task blocks (one of each load) separated by baseline periods (4 s blank), with 10 trials of the same load grouped into one task block (Figure 1A). The order of task blocks was randomized for each task run. Data from a total of 20 trials (i.e., 2 runs) of each task load were used for each participant. Individual accuracy (proportion of correct trials out of total trials) and average response time (for correct trials) for each task type were used as task performance measures across both age groups.

### Image Acquisition

Resting-state and SWM task fMRI data were collected for each participant using a 3T MRI scanner (Siemens, Erlangen, Germany) at the Centre for Translation MR Research, National University of Singapore. During resting state, participants fixated on a cross presented on the center of the screen. They were asked to keep their eyes open and not to fall asleep or think of anything in particular. The scanner underwent an upgrade (from Tim Trio to Prisma Fit) in the midst of data collection, resulting in all young adults from the first dataset and 21 older adults scanned before the upgrade, while 13 older adults were scanned after the upgrade. This variation in scanner was used as a nuisance covariate in analyses for the older adults. All functional scans were matched in key parameters (TR/TE = 2,000/30 ms, voxel size = 3.0 × 3.0 × 3.0 mm^3^, FOV = 192 × 192 mm^2^, 36 axial slices, flip angle = 90°, bandwidth = 2112 Hz/pixel before upgrade, 2298 Hz/pixel after upgrade). There were 235 volumes of resting-state fMRI and about 170 × 2 (runs) volumes of task fMRI data per participant for subsequent preprocessing and analyses.

High-resolution T1 structural MRI scans were also obtained for all participants using magnetization-prepared rapid gradient echo sequence (MPRAGE) with matched imaging parameters across the two scanner systems (before upgrade: TR/TE = 2,300/2.98 ms, voxel size = 1.0 × 1.0 × 1.0 mm^3^, FOV = 256 × 240 mm^2^, 192 sagittal slices, flip angle = 9°, bandwidth = 240 Hz/pixel; after upgrade: TR/TE = 2,300/2.28 ms, voxel size = 1.0 × 1.0 × 1.0 mm^3^, FOV = 256 × 240 mm^2^, 192 sagittal slices, flip angle = 8°, bandwidth = 200 Hz/pixel).

### Image Preprocessing

Preprocessing of the structural and functional MRI data was done using FSL (Jenkinson, Beckmann, Behrens, Woolrich, & Smith, 2012) and AFNI (Cox, 1996), following standard protocols in our previous work (Liu et al., 2018; K. K. Ng et al., 2016). In brief, structural images were preprocessed by reduction in image noise (SUSAN), skull stripping (Brain Extraction Tool), linear and nonlinear registration to the Montreal Neurological Institute (MNI) 152 standard space (FLIRT and FNIRT), and brain segmentation into gray matter, white matter, and cerebrospinal fluid (CSF) compartments. Functional images were preprocessed by removing the first five volumes, slice time correction, motion correction, skull stripping, spatial smoothing with Gaussian kernel (6-mm full width at half maximum), temporal band-pass filtering (0.009–0.1 Hz), removal of first and second-order trends, coregistration with structural image (Boundary-Based Registration), nonlinear registration to standard space (FNIRT), and regression of nuisance signals (CSF, white matter, global signal, and six motion parameters). Global signal was regressed to decrease effects of nuisance signals and positive bias in FC measures (Hayasaka, 2013; Power et al., 2014) and improve associations between FC measures and task performance (Li et al., 2019). For consistency, the same preprocessing steps were performed for all rest and task fMRI scans across young adult and older adult datasets except band-pass filtering and detrending, which were only performed for rest fMRI data.

Functional images were subjected to quality control for motion (maximum absolute displacement ≤ 4 mm and maximum framewise displacement ≤ 2 mm), and participants with excessive head motion were excluded. Visual inspection was also performed for coregistration of functional to structural images for each fMRI scan for each participant. Mean relative motion for rest and task and mean absolute motion for task was higher in older adults compared to young adults (two-sample *t* ≥ 2.94, *p* ≤ 0.004), while mean absolute motion for rest was not significantly different between young and older adults (two-sample *t* = 1.97, *p* = 0.05). Mean relative motion was thus used as a nuisance variable in statistical analyses to control for the effects of motion. To further ensure that differences in motion parameters between age groups did not account for age-related changes, validation analyses were performed with a subset of 66 young adults with no differences in mean relative and absolute motion in both rest and task from older adults (all two-sample *t* ≤ 1.97, *p* > 0.05).

### Derivation of FC Similarity

Consistent with previous work on rest-task FC similarity (Schultz & Cole, 2016; Zuo et al., 2018), task activations were regressed from task fMRI data before task FC was derived (Cole et al., 2014) since this has been shown to reduce false positives in task FC estimates (Cole et al., 2019) and improve task FC reliability (Cao et al., 2014). Specifically, main and derivative regressors were created for dot presentation (single boxcar regressor), maintenance, probe, response, fixation between trials, and correct and wrong trials, all of which were convolved with a canonical hemodynamic response function. To obtain load-specific FC for SWM load, blocks of the same task load were concatenated to form load-specific task fMRI data, with all baseline periods discarded.

To extract time series from preprocessed fMRI data, we used a parcellation scheme comprising 400 cortical regions of interest (ROIs) (Schaefer et al., 2018) and 30 subcortical ROIs (Choi, Yeo, & Buckner, 2012; Tzourio-Mazoyer et al., 2002) grouped into nine networks (executive control, default mode, dorsal attention, limbic, salience/ventral attention, somatomotor, temporal-parietal, visual, and subcortical networks). FC between each pair of ROIs was quantified for each participant by taking the Pearson’s correlation of their fMRI time series. FC matrices for rest and each of the three task loads were then constructed for each participant using the correlation z-values after Fisher’s r-to-z transformation. ROIs were arranged according to their previously defined functional network assignments (Schaefer et al., 2018) such that cells along the main diagonal represented FC between ROIs of the same network (intranetwork), while off-diagonal cells represented FC between ROIs from different networks (internetwork).

Brain functional reconfiguration was measured by both global and network-level FC similarity. Global FC similarity was computed by taking Pearson’s correlation (r-to-z transformed) of the individual-level vectorized whole-brain FC matrices (considering only the lower triangular entries) between two conditions. This was separated into rest-task FC similarity and stepwise FC similarity (Figure 1B). Rest-task FC similarity was computed by taking the correlation between rest FC and the three load-dependent task FC matrices (i.e., at dot1, dot3, dot5). Stepwise FC similarity was computed by taking the correlation between each pair of FC matrices corresponding to a higher task load and the next (lower) load (i.e., dot1 vs. rest, dot3 vs. dot1, dot5 vs. dot3).

For network-level reconfiguration, FC similarity was computed for intranetwork and internetwork components separately for all SWM tasks. Intranetwork FC similarity was calculated using only the diagonals of the FC matrices (FC from ROIs within the same network), while internetwork FC similarity was calculated using the off-diagonals (FC from ROIs of each network to all other networks). This yielded one intranetwork and one internetwork FC similarity value for each network. Pearson’s correlation (r-to-z transformed) was also used for network-level FC similarity computations and both rest-task and stepwise FC similarity were calculated.

### Statistical Analysis

To test for age and task load differences, as well as their interactive effects on SWM task performance and FC similarity, analysis of variance (ANOVA) tests were performed across young and older datasets using the following equation:

$$Y∼Age\;\text{group}*\text{Load}+Sex+\text{Task}\;\text{motion}+\text{Rest}\;\text{motion}+\text{Scanner}+\left(1|\text{Subject}\right)$$ (1)

where *Y* was the SWM accuracy, SWM response time, rest-task FC similarity, or stepwise FC similarity. Separate models were used for each type of FC similarity for whole brain and for each network. *Age group*, *Sex*, and *Scanner* were binary dummy variables, while *Load* was a categorical variable representing the different task loads or load pairs. *Rest motion* and *Task motion* were the mean framewise displacement (FD) during the rest and task fMRI scan, respectively. For stepwise FC similarity, *Rest motion* was set to zero for the load pairs involving two task loads. For post hoc analyses, Tukey’s honest significant differences (HSD) tests were run in R (R Core Team, 2018). For network-level analyses, Bonferroni correction for multiple comparisons was applied on the *p* values from the main effects from the ANOVA models at the level of *p* < 0.05 based on number of networks (*α* = 0.05/9 ≃ 0.006).

To test for associations between global FC similarity and behavior for each dataset and SWM task, we performed partial Spearman correlations between global FC similarity and SWM task performance, controlling for age, gender, and mean task FD within each dataset. For rest-task FC similarity, mean rest FD was also regressed during the partial correlation analyses. For older adults, scanner difference was also included as a covariate. FC similarity was correlated with SWM accuracy and response time at the corresponding task load; for stepwise FC similarity, the task performance measure at the higher task load was used (e.g., dot1-dot3 FC similarity with dot3 accuracy). We reported results at the level of uncorrected *p* < 0.05 for these analyses. Bootstrap analyses (10,000 iterations) were performed to obtain standard error estimates for the partial Spearman correlation values for visualization.

To evaluate network-level contributions to the significant associations at the global level, we performed partial Spearman correlations as described above for SWM task performance with intra- and internetwork FC similarity. Bonferroni correction for multiple comparisons was applied on the *p* values from the partial correlation analyses at the level of *p* < 0.05 based on number of networks (*α* = 0.05/9 ≃ 0.006). All statistical analyses were performed in MATLAB 2015a (The MathWorks, Inc., Natick, Massachusetts, United States) and R 4.3.1 unless otherwise stated. Graphs were created using the ggplot2 package (Wickham, 2009) in R (R Core Team, 2018).

## RESULTS

### Age and Load Effects on SWM Task Performance

**Figure 2.**
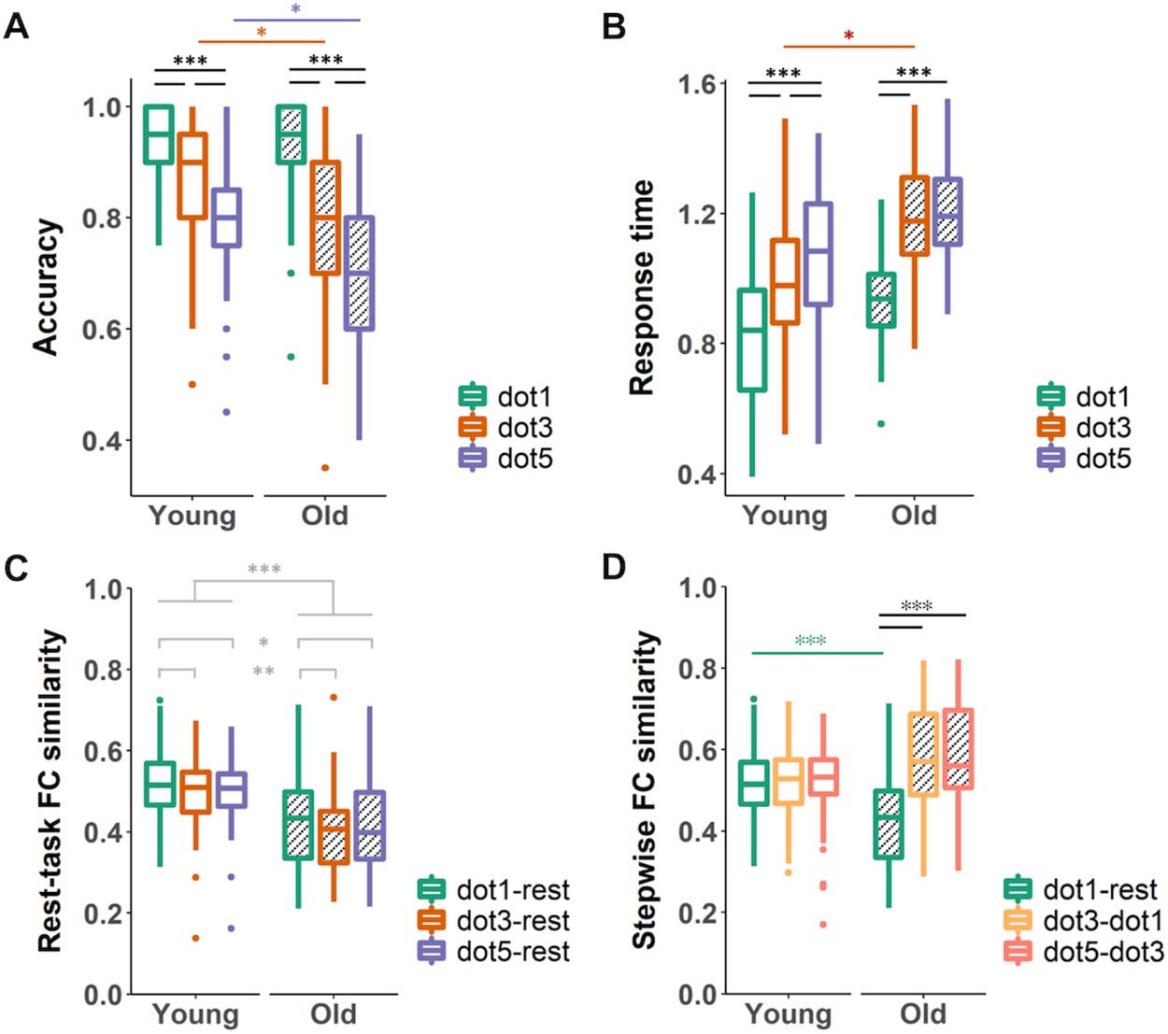
Task performance and global functional reconfiguration showed age and load effects. Age and load interactions were observed in accuracy (A) and response time (B) in the spatial working memory (SWM) task. Larger decreases in SWM accuracy and increases in response time were observed with higher task load in older adults compared to young adults. (C) No interaction between age and task load were found for global rest-task FC similarity. Age effects (lower FC similarity in older compared to young adults) and load effects (higher FC similarity at low load compared to intermediate and high loads) were observed. (D) Global stepwise FC similarity showed age and load interaction effect, with only older adults having lower FC similarity between rest and task (dot1) compared to between task loads. Asterisks indicate significant differences using Tukey’s honest significant differences test on post hoc contrasts; *p < 0.05, **p < 0.01, ***p < 0.001.

We found effects of age group and task load on both behavioral and functional brain measures. Specifically, the ANOVA analysis revealed significant age and load interaction effects for SWM accuracy (*F*_2,420_ = 9.15, *p* < 0.001) and SWM response time (*F*_2,420_ = 7.68, *p* = 0.001), such that larger decreases in task performance with increased load were observed in older compared to young adults (Figure 2A and 2B). Post hoc analyses revealed that in general, SWM accuracy decreased and response time increased with load in both young and older adults, with worse performance in older adults (Supporting Information Table S1). These findings are consistent with previous studies on SWM tasks (Boonstra, Powell, Mehrkanoon, & Breakspear, 2013; Gevins, Smith, McEvoy, & Yu, 1997; Kessels, Meulenbroek, Fernández, & Olde Rikkert, 2010; Proskovec, Wiesman, Heinrichs-Graham, & Wilson, 2019; Roux, Wibral, Mohr, Singer, & Uhlhaas, 2012).

### Age and Load Effects on Global FC Similarity

For global rest-task FC similarity, age (*F*_1,419_ = 15.22, *p* < 0.001) and load effects (*F*_2,420_ = 4.22, *p* = 0.015) were found, but not interaction effects (*F*_2,420_ = 0.23, *p* = 0.798). Rest-task FC similarity was significantly lower in older compared to young adults (Tukey’s HSD *p* < 0.001), while rest FC was more similar to task FC at low load compared to intermediate (Tukey’s HSD *p* = 0.003) and high loads (Tukey’s HSD *p* = 0.046; Figure 2C).

On the other hand, age-by-load interaction effects were found for global stepwise FC similarity (*F*_2,420_ = 40.37, *p* < 0.001). Post hoc analyses showed that stepwise FC similarity was more ‘load-dependent’ in older compared to young adults: while it was comparable among all transitions from rest to the highest load in the young, older adults showed higher FC similarity when the transition was between different task loads (dot3 vs. dot1 and dot5 vs. dot3) than when it was between rest and task (dot1 vs. rest). The FC similarities between rest and easy load (dot1 vs. rest) was also lower than that of the young adults (Figure 2D, Supporting Information Table S2).

### Age and Load Effects on Network FC Similarity

**Figure 3.**
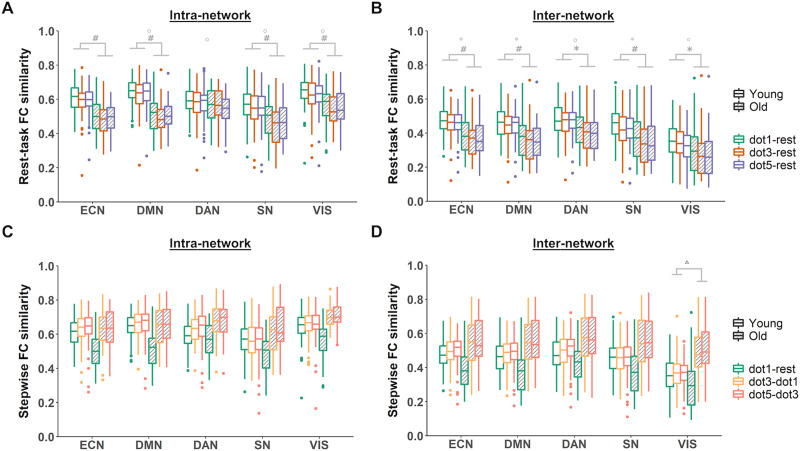
Network-level functional reconfiguration showed age effects. For FC similarity from rest to task, higher order cognitive networks and task-relevant networks showed age effects in intra network connections (A) and both age and load effects in internetwork connections (B). Asterisks indicate significant age group differences, *p < 0.05, **p < 0.01, while circles (○) indicate significant load differences at *p* < 0.05. Hashes (#) and double circles (◎) indicate age and load effects that survived Bonferroni correction for multiple comparisons (*α* = 0.05/9 ≃ 0.006), respectively. For stepwise FC similarity, all networks showed age × load interactive effects after adjusted for multiple comparisons (C–D), typically showing larger FC similarity differences between rest-task transition (dot1-rest) and the task load transitions (dot3-dot1 and dot5-dot3) in the older adults compared to young adults. Triangle (▵) indicates that older adults had higher FC similarity during task load transitions than young adults (red and orange bars) in the visual network (VIS), a pattern not observed in other displayed networks. Only a subset of representative networks are illustrated; see Supporting Information Table S3 (rest-task) and Supporting Information Table S4 (stepwise) for all statistics on age, load and interaction effects.

At the network level, rest-task FC similarity yielded similar patterns as its global counterparts in both intra- and internetwork profiles. Specifically, several associative networks (DMN, ECN, SN) and visual network showed significant main effects of age and/or load in both intra- and internetwork FC similarity (Supporting Information Table S3). We note that all main effects of load in intranetwork FC similarity did not survive multiple comparison correction, while the load effects in internetwork FC similarity of ECN, DMN, and SN remained after such adjustment. In general, (1) older adults had significantly lower rest-task FC similarity than young adults, and (2) rest FC was more similar to task FC at low load (dot 1 vs. rest) compared to intermediate load and/or high loads (Figure 3A and 3B), similar to the global load effect.

For stepwise FC similarity, all networks yielded significant age-by-load interactions in both intra- and internetwork similarity after Bonferroni adjustment (Supporting Information Table S3). This presence of interactive effects was consistent with that at the global level, but the exact patterns differed across networks and could be summarized into several kinds (Figure 3C and 3D; full statistical summary in Supporting Information Table S4). First, in all but limbic and subcortical networks, both intra- and internetwork FC similarities yielded similar patterns as the global stepwise FC similarity, namely that (1) the FC similarity of rest-task transition (dot1 vs. rest) was lower in older adults compared to young adults, and (2) for older adults, the FC similarity of this transition was significantly lower (i.e., less efficient reconfiguration) than the FC similarities within task load transitions (dot3 vs. dot1 and dot5 vs. dot3). Second, older adults had *higher* intra- and/or internetwork FC similarities (i.e., more efficient reconfiguration) between task load transitions (dot3 vs. dot1 and dot5 vs. dot3) than younger adults in the somatomotor, limbic, subcortical, and visual networks. Third, unlike the global stepwise results, young adults also showed load-modulated FC similarity in the DAN, DMN, ECN, limbic, and subcortical networks, such that the FC similarity of rest-task transition (dot1 vs. rest) was significantly lower (i.e., less efficient reconfiguration) than either the highest load transition only (dot5 vs. dot3) or both the highest and intermediate load transitions (dot3 vs. dot1). Representative results were illustrated in Figure 3C and 3D. Overall, these findings indicate that network-level FC similarity contributed to the age and load differences in global FC similarity, particularly for higher order and task-relevant networks.

### Association Between FC Similarity and SWM Task Performance Involves Higher Order and Task-Relevant Networks in Young Adults

**Figure 4.**
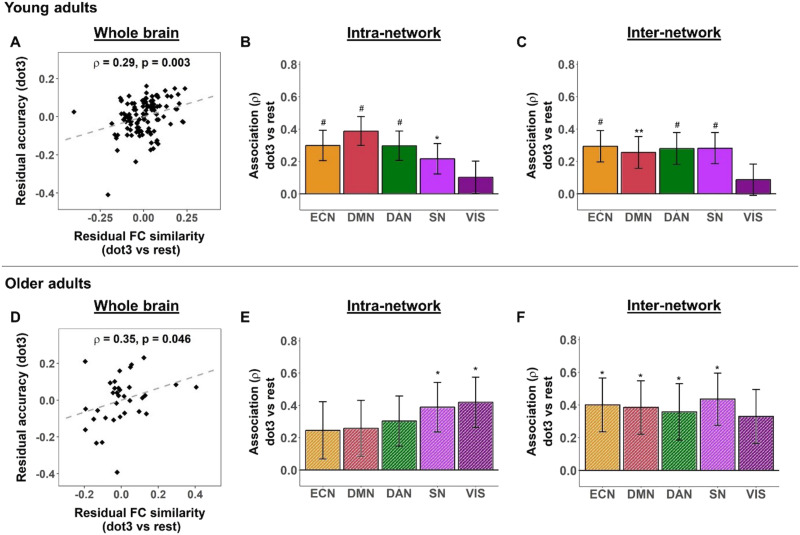
More efficient rest-task functional reconfiguration is associated with better task performance in spatial working memory (SWM)-load task in young and older adults. Higher global functional connectivity (FC) similarity between rest and intermediate load was correlated with better SWM accuracy in SWM-load task in young (A) and older adults (D). Each dot in the scatter plots represents one participant. For young adults, better accuracy in SWM-load task was associated with higher intranetwork (B) and internetwork (C) rest-task FC similarity for higher order and task-relevant networks. For older adults, better accuracy in SWM-load task was related to higher network-level rest-task FC similarity between (F) but not within (E) higher order cognitive networks. *ρ* values represent Spearman’s correlation between global FC similarity and SWM task performance controlled for age, gender, and mean FD for rest and task (and scanner difference for older adults). Error bars indicate standard error estimated by bootstrap analyses. Asterisks indicate significant correlations (*p < 0.05, **p < 0.01). Hashes (#) indicate correlations that survived Bonferroni correction for multiple comparisons over networks (*α* = 0.05/9 ≃ 0.006); no multiple comparisons corrections were performed for associations with global FC similarity. Only selected networks are illustrated; see Supporting Information Table S5 for all correlations.

We found a positive relationship between global FC similarity and SWM task performance in both rest-task and stepwise comparisons (Figure 4, left), such that higher global FC similarity was generally associated with better SWM accuracy. Specifically, this positive association was found for FC similarity between rest and the easy load (dot1 vs. rest) and SWM accuracy at the easy load (dot1), FC similarity between rest and the intermediate load (dot3 vs. rest) and SWM accuracy at the intermediate load (dot3), as well as FC similarity between the east and intermediate load (dot3 vs. dot1) and SWM accuracy at the intermediate load (dot3).

Consistent with results from the global FC similarity analyses, we found a positive relationship between intranetwork and internetwork FC similarity and SWM accuracy for the rest-task (Supporting Information Table S5) as well as stepwise (Supporting Information Table S6) comparisons. These associations consistently involved task-relevant dorsal attention network (DAN), higher order cognitive networks (ECN and DMN), and SN (Figure 4, middle, right), most of which survived Bonferroni correction for multiple comparisons (*α* = 0.05/9 ≈ 0.006).

No association with SWM accuracy was found for FC similarity involving the high load (dot5) for both rest-task and stepwise comparisons at the global level (all uncorrected *p* > 0.05; Supporting Information Table S5 and S6), and network-level associations were thus not explored.

### Association Between Rest-task FC Similarity and SWM Task Performance Relies More on Internetwork Contributions in Older Adults

**Figure 5.**
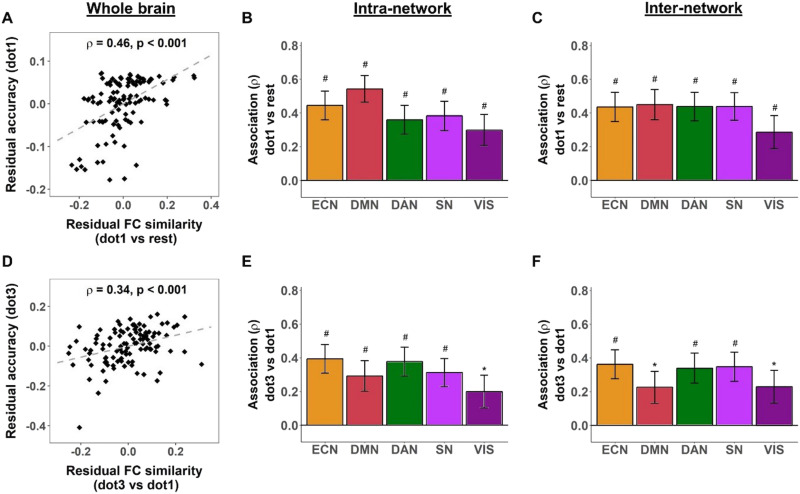
More efficient stepwise reconfiguration associates with better spatial working memory (SWM)-load task performance only in young adults. Higher global stepwise functional connectivity (FC) similarity was correlated with better SWM accuracy in SWM-load task only in young adults, from rest to easy load (A) and from easy to intermediate load (D). Each dot in the scatter plots represents one participant. Better accuracy in SWM-load task was associated with higher intranetwork (B) and internetwork (C) FC similarity from rest to easy load for higher order and task-relevant networks. Better accuracy in SWM-load task was also related to higher intranetwork (E) and internetwork (F) FC similarity between easy and intermediate loads for higher order and task-relevant networks. *ρ* values represent Spearman’s correlation between global FC similarity and SWM task performance controlled for age, gender, and mean FD for task (and mean FD for rest for panels A–C). Error bars indicate standard error estimated by bootstrap analyses. Asterisks indicate significant correlations (*p < 0.05). Hashes (#) indicate correlations that survived Bonferroni correction for multiple comparisons over networks (*α* = 0.05/9 ≃ 0.006); no multiple comparisons corrections were performed for associations with global FC similarity. Only selected networks are illustrated; see Supporting Information Table S6 for all correlations.

Positive associations between rest-task FC similarity and SWM accuracy were also observed in older adults (Figure 5; Supporting Information Table S5), but not for stepwise FC similarity (Supporting Information Table S6). Specifically, the associations involved FC similarity between rest and the intermediate load (dot3 vs. rest) and SWM accuracy at the intermediate load (dot3). This positive association was first found for global FC similarity, but associations at the network level did not survive multiple comparisons correction (Supporting Information Table S5).

Given that the hypothesis of positive associations between FC similarity and behavior based on the literature was aligned with the results from the young adult dataset in this study and at the whole-brain level in older adults, and noting the smaller sample size of the older adult dataset, we considered the uncorrected *p* values to explore the contribution of network-level FC similarity to the global association for this load (dot3 vs. rest) in older adults. Using uncorrected *p* values (*α* = 0.05), we found that network-level associations consistently involved the contributions of SN and VIS across intranetwork and internetwork levels (Figure 5, middle and right). At the internetwork level, additional networks (ECN, DMN, and DAN) showed positive associations between FC similarity and SWM accuracy, similar to the network contributions in young adults.

No other associations with SWM task performance and FC similarity were found in older adults at the global level (all uncorrected *p* > 0.05; Supporting Information Table S5 and S6), and network-level associations were thus not explored.

### Validation Analyses

To examine if differences in FC similarity may be partly explained by higher topological variability in older adults compared to young adults during task, we computed ‘within-load’ FC similarity by deriving task FC matrices for each task load and run separately and then correlated the two FC matrices of the same load. Subjecting these within-load reconfiguration values to a linear mixed model (treating within-condition similarity as the dependent variable in Equation 1) did not yield any significant age group (*p* = 0.88) or age-by-load interaction effects (*p* = 0.13), suggesting that aging may not alter the stability of brain functional configurations in such a short timescale, and a difference in this stability is unlikely to fully explain the lower rest-task similarity with aging.

To examine if chartering the transition between ‘brain states’ (FC similarity) provided unique insights beyond functional connectivity strength in a state per se, further validation analyses were conducted with task FC instead of rest-task FC similarity (Supporting Information Table S7). Briefly, after deriving the FC matrices for each task load (see subsection Derivation of FC Similarity of Methods), the cells (FC edges between a pair of regions) of each matrix were averaged either globally or according to the network membership of the region pairs into measures of intranetwork FC (e.g., both regions belonging to the DMN) and internetwork FC (e.g., one region from the DMN and the other from one of the reminder networks), yielding 9 networks × 3 edge types (whole brain or intra- vs. internetwork) × 3 loads FC measures per participant. Using these values as our variables of interest, no significant associations were found at any task load for either age group at the global level. For young adults, network-level averaged FC at both intra- and internetwork levels also did not show any significant associations with task performance after multiple comparisons correction. These suggest that looking at changes between rest and task using the FC similarity metric provides additional value for understanding behavior beyond the original rest and task FC metrics alone. For older adults, ECN, DMN, DAN, and SN had significant positive associations (uncorrected *p* values < 0.05) for FC with SWM accuracy, particularly for task FC, at the internetwork level. These indicate that stronger FC between networks was related to better task performance, further supporting the pattern of reduced functional segregation in aging as observed with the FC similarity measure.

To ascertain that age-related differences in motion could not account for our results, additional validation analyses were conducted in a subset of young adults motion matched to older adults, which resulted in similar results for global and network-level analyses of FC similarity (Supporting Information Table S8 and S9). In addition to accuracy, we also performed association analyses with SWM response time as the task performance measure. However, global FC similarity showed no correlation with response time in both young and older adults at any load (all uncorrected *p* > 0.05), and network-level associations were not further explored. To ascertain our findings could not be attributed to possible age-related differences in task execution (e.g., speed-accuracy trade off), we also performed an additional set of analyses controlling for response time on our main analyses and found similar results as reported in subsections Age and Load Effects on Network FC Similarity and Association Between FC Similarity and SWM Task Performance Involves Higher Order and Task-Relevant Networks in Young Adults of Results (Supporting Information Table S10 and S11).

Finally, as a first step to demonstrate the generalizability of our findings to different WM tasks, we repeated the same FC similarity analyses in two other variants of SWM tasks in young adults (Supporting Information Methods). The same positive associations between rest and task FC similarity and SWM task performance, mainly involving the ECN and DAN, were found (Supporting Information Results and Table S12). These validation results lend further support to the generalizability of the close link between rest-task FC similarity and performance across different working memory tasks.

## DISCUSSION

In this study, we aimed to investigate how the behavioral relevance of global and network-specific functional reconfiguration differs across task loads and age groups. At both global and network level, we found that older adults and higher task loads were associated with more functional reconfiguration from rest to task when comparing the functional topology between rest and varying task loads. Across many brain networks, older adults also reconfigured more than young adults as task demands increased progressively from rest into higher task loads. Importantly, more efficient reconfiguration in higher order brain functional networks was consistently related to better performance in both young and older adults at the global as well as intra- and internetwork levels. Specifically, more efficient rest-task and stepwise functional reconfiguration (i.e., less reconfiguration) were associated with better task performance in young adults, consistently involving higher order and visuospatial task-relevant networks. However, in older adults, functional reconfiguration was only relevant from rest to task and involved mostly internetwork reconfiguration, while intranetwork reconfiguration of higher order cognitive networks was not as behaviorally relevant. These patterns suggest that optimal brain configurations for task performance may differ in young and older adults, with older adults likely to recruit additional functional networks, some of which potentially serving as a compensatory mechanism. Our findings thus highlight the relevance of brain functional reconfiguration to the study of cognitive aging.

### Functional Reconfiguration From Rest to Task and Between Task Loads Is Less Efficient in Aging

Overall, compared to young adults, we found that older adults generally evidence more functional network reconfiguration on both global and network levels from rest to task. At the network level, high-order cognitive networks (default = DMN, executive control = ECN, and to a lesser extent dorsal attention = DAN and salience = SN) and the visual network (VIS) showed the most consistent effects both at intra- and internetwork connections. Furthermore, the ECN, DMN, and SN demonstrated more functional reconfiguration as a function of task load especially in their internetwork connections. Stepwise network reconfiguration painted a more complex picture with the presence of age-by-load interactions. At the global level and across most networks, older adults had notable reconfiguration from resting to task state (i.e., lower rest to dot 1 FC similarity than other transitions from low to high load, which is also lower than that of young adults), but task load transitions (i.e., dot 1 to 3 & dot 3 to 5) did not induce detectable reconfiguration. Young adults also showed similar patterns, but the rest-task reconfiguration effect size (rest-dot1 transition vs. task-load transitions) was either not statistically significant or much less substantial than that in the older adults (e.g., ECN). Notable exceptions were observed for the somatomotor (SMN, intra- and internetwork), limbic (LN, intra- and internetwork), visual (VIS, internetwork) and the subcortical (SUB, internetwork) networks, in which older adults interestingly evidence *less* reconfiguration between lower and higher task loads compared to their young counterparts. Therefore, ‘high-order’ functional brain networks appear to yield a consistent pattern of more reconfiguration in older adults (especially from rest to easy load) and higher task loads (at least when compared to rest), while it is more variable among lower order brain functional networks.

Pioneering work on rest-task functional network reconfiguration on young adults argued that more efficient network updating may result from the functional organization being more ‘task-ready’ during less goal-oriented conditions such as the resting state (Schultz & Cole, 2016). Our results of more reconfiguration from rest-to-task networks in the older adults may suggest that (1) the resting-state organization of older adults cannot be tuned to be task-ready as successfully as young adults, possibly owing to age-related compromises in neural signal transmission and functional organization (Naik, Banerjee, Bapi, Deco, & Roy, 2017; Reuter-Lorenz & Park, 2014) appearing as, for instance, lower network segregation and distinctiveness (Chong et al., 2019; Setton et al., 2022), and (2) consistent with the CRUNCH hypothesis (Reuter-Lorenz & Cappell, 2008), older adults may resort to the recruitment of additional brain networks starting at low cognitive loads for normal functioning, exhausting neural resources that are only deployed for higher cognitive demands in younger individuals (Prakash et al., 2009; Reuter-Lorenz & Mikels, 2006). These early and additional recruitments may manifest as more substantial initial changes in functional topology, but not particular sensitivity to subsequent task loads.

The pattern discussed above was present in most ‘high-order’ cognitive networks (such as DMN and ECN) and ‘task-positive’ networks (such as DAN), whose connectivity alterations have been regularly shown to be prominent features of neurocognitive aging (Chan, Park, Savalia, Petersen, & Wig, 2014; Liem, Geerligs, Damoiseaux, & Margulies, 2021; Spreng & Turner, 2019). Interestingly, the ‘lower order’ networks, namely somatomotor, limbic, visual, and subcortical networks yielded a different pattern that characterized older adults with more efficient network reconfiguration than young adults during task. While we do not have an a priori hypothesis, these networks encompass the basal forebrain, basal ganglia, and the visual system, which are essential for mediating arousal and motivation (Bell & Shine, 2016). Sustained consistent network configuration (high FC similarity, low reconfiguration) in these networks by older adults might indicate an enhancement of these processes for them to complete the SWM task competently (Mather, 2020), albeit in a less flexible manner.

### High Performers Have More Efficient Functional Reconfiguration in Both Young and Older Adults

Our findings demonstrate that the positive association between more efficient network reconfiguration and better cognitive ability found in previous studies conducted on young adults (Schultz & Cole, 2016; Zuo et al., 2018) also generalizes to older adults. As hypothesized, more efficient reconfiguration (higher rest-task FC similarity) was associated with better performance in both young and older adults, indicating that high performers, regardless of age, are those who can update their brain functional organization efficiently to meet task demands. This was also true across different SWM task types (SWM-D, SWM-I, and SWM load) in young adults. These findings support Schultz and Cole (2016)’s proposal that individuals with intrinsic FC configurations that are well poised to transition into task configurations are able to perform better in tasks as a generalizable observation.

### Association Between Task Performance and Network Reconfiguration Is Supported by Similar Networks Across SWM Loads

Consistent with our hypothesis, more efficient functional reconfiguration of higher order cognitive networks and visuospatial task-relevant networks were consistently involved in the positive association with SWM performance, contributing to the positive associations observed at the global level. Generally, more efficient ECN, DMN, DAN, and SN reconfiguration (higher FC similarity) at both intra- and internetwork levels were related to better task performance, particularly in young adults. These findings are in line with established functions supported by these networks, since ECN is important for executive functions and externally oriented goals (Seeley et al., 2007; Turner & Spreng, 2015), while DAN is particularly important for SWM (Courtney, Petit, Maisog, Ungerleider, & Haxby, 1998; Leung, Oh, Ferri, & Yi, 2007). Depending on task requirements, DMN activity is either suppressed (Fox et al., 2005; Raichle et al., 2001) or engaged in specific processes (Smith, Mitchell, & Duncan, 2018) during externally directed tasks, with WM demands modulating DMN FC suppression (Liang, Zou, He, & Yang, 2016) and connectivity with other networks (Bluhm et al., 2011).

Furthermore, more efficient SN reconfiguration was also related to better task performance in both young and older adults. SN is responsive to changes in WM load and its activity is related to WM capacity, explaining the relevance of SN reconfiguration to SWM accuracy (Engström, Landtblom, & Karlsson, 2013).

Reconfiguration of VIS was also behaviorally relevant, particularly in older adults, which may be related to changes in level of visual attention (Kwon, Watanabe, Fischer, & Bartels, 2017; Spadone et al., 2015) and mechanisms of visual imagery supporting WM maintenance (Albers, Kok, Toni, Dijkerman, & de Lange, 2013; Keogh & Pearson, 2011; Santangelo & Bordier, 2019).

### Association Between Task Performance and Network Reconfiguration Is Supported by Fewer Networks and Less Responsive to Increased Task Demands in Aging

Importantly, our findings indicate that behaviorally relevant associations at the intranetwork level involved fewer networks in older adults compared to the internetwork level (SN and VIS), while similar networks were involved at both intra- and internetwork levels in young adults. This could possibly be due to the reduced functionality within networks with aging (Betzel et al., 2014; Geerligs, Maurits, et al., 2014; K. K. Ng et al., 2016; Spreng et al., 2016). In particular, ECN, DMN, and DAN consistently showed associations of higher network-level FC similarity with better SWM accuracy in young adults. However, associations involving these networks appeared not to be statistically significant in older adults. As mentioned above, ECN, DMN, and DAN support functions relevant to the SWM task. The activities of ECN and DAN have been shown to increase with higher memory load (Liang et al., 2016; Todd & Marois, 2004), but are less responsive to SWM load in older adults compared to young adults (Nagel et al., 2009). The increased deactivation of DMN at higher task demands is also impaired in older adults (Park, Polk, Hebrank, & Jenkins, 2010). Taken together, these could explain our observations that more efficient ECN, DMN, and DAN intranetwork reconfiguration was more relevant to SWM performance in young adults. Of note, the difference in sample size between the two groups (*N* = 34 for older adults and *N* = 109 for young adults) could have contributed to this difference, and future work with more balanced sample sizes would be needed to validate this finding.

What remains consistent between the two age groups is that more efficient internetwork functional reconfiguration of ECN, DMN, DAN, and SN underlies better behavioral performance. Indeed, as summarized in the previous section, the internetwork FC similarities of these networks between rest and task were those that yielded statistically significant load effects. SN has been proposed as a mediating hub that coordinates resource allocation to different functional networks for performance of cognitive tasks (Camilleri et al., 2018), particularly for switching between ECN and DMN (Menon & Uddin, 2010; Sridharan, Levitin, & Menon, 2008). Furthermore, FC of SN with ECN and DMN was found to increase to meet higher cognitive demands of increased WM load (Liang et al., 2016). Increases in FC of DAN with ECN during WM task was also correlated with WM improvement after training in a previous study (Thompson, Waskom, & Gabrieli, 2016). These reports support our findings that the reconfiguration of internetwork FC of these higher order and SWM-related networks are behaviorally relevant.

Based on the inter- and intranetwork results, we speculate that aging may be characterized by a ‘task-general’ state that sees a shift in importance from intranetwork to internetwork reconfiguration in terms of relevance to a given cognitive domain. This is consistent with reports of reduced functional segregation of functional networks in aging literature (Chan et al., 2014; Chong et al., 2019; K. K. Ng et al., 2016). In particular, intranetwork FC of DMN has been found to decrease with age, often accompanied by increases in FC of DMN with attentional and fronto-parietal networks (Andrews-Hanna et al., 2007; Damoiseaux et al., 2008; Ferreira et al., 2016; Grady, Sarraf, Saverino, & Campbell, 2016; K. K. Ng et al., 2016; Sambataro et al., 2010; Spreng et al., 2016). Future work could use a variety of tasks during fMRI scans to obtain this task-general reconfiguration in older adults, averaging the FC similarity over the tasks to study the task-general patterns (Cole et al., 2014; Schultz & Cole, 2016).

Collectively, this shift from intra- to internetwork of behavioral relevance in functional architecture and the putative processes underlying the cohort differences in this architecture (e.g., CRUNCH and arousal modulation in subsection Functional Reconfiguration From Rest to Task and Between Task Loads Is Less Efficient in Aging of Discussion) may appear to qualify as a compensation by additional recruitment form of compensatory mechanisms in aging (Cabeza et al., 2018). However, this seem to be at odds with the age group invariant positive correlations, since to qualify as a compensatory process, one would expect *more* network updates to be associated with better SWM accuracy among the older adults (Cabeza et al., 2018; Eyler, Sherzai, Kaup, & Jeste, 2011). Indeed, the cohort-level difference may be more parsimoniously explained by age-related reduction in neural efficiency, necessitating an average older adult to devote more resources even at low cognitive demand compared to an average young adult (Cabeza et al., 2018; Rypma, 2007). It then follows that older individuals who can sustain similar functional assemblies across different situations, despite it having an altered topology (toward network cross-talks) and being less optimal than the young adult’s version (Naik et al., 2017), may imply preserved neural resources or processing efficiency, thus maintaining a performance edge over their peers. In this sense, efficient network reconfiguration per se in older adults may be better understood as a noncompensatory phenomenon such as reserve (Cabeza et al., 2018). Existing studies on age-related compensation, maintenance, and reserve mechanisms seldom consider network reconfiguration concurrently, further studies elucidating the interaction between age-related evolution in functional topological landscape (Ezaki, Sakaki, Watanabe, & Masuda, 2018; Naik et al., 2017) and these strategies may unravel novel principles governing functional adaptations. For instance, Hennessee et al. (2022) reported that middle-aged adults with better performance during a semantic judgment task were those who maintained ‘youth-like’ prefrontal lateralization, while among older adults they were those with more bilateral prefrontal activation, which may reflect a qualitative shift along the ‘developmental timing of compensatory brain activity’ as proposed in the STAC-r model of aging (Reuter-Lorenz & Park, 2014). One potential modulator of this shift could be one’s ability to maintain a task-ready state across conditions.

Interestingly, more efficient functional reconfiguration in both rest-task and stepwise conditions was associated with better task performance in young adults, while only more efficient rest-task functional reconfiguration was associated with better task performance in older adults. This observation that stepwise reconfiguration was behaviorally relevant in young adults, but not older adults, could reflect the reduced capability of older adults to change their functional connections in response to increasing task loads as we alluded to earlier in subsection Functional Reconfiguration From Rest to Task and Between Task Loads Is Less Efficient in Aging of Discussion. Furthermore, we found no global association between functional reconfiguration to the difficult task load (from rest or other loads) with SWM performance in either age group. We speculate that due to the difficulty of this task load, which required participants to remember the position of five dots (at the upper limit of typical WM capacity (Cowan, 2001, 2010)), the rest-task functional reconfiguration measure used in this study may not sufficient to capture the performance on the task. Complex strategies may have been used by participants for the difficult task load (McNamara & Scott, 2001) such that more fine-grained functional measures may be needed to delineate the interindividual differences in task performance.

### Limitations and Future Work

We note a few limitations of the current study. First, this study investigated the behavioral relevance of functional reconfiguration in aging using only SWM tasks. Although our results were convergent with similar studies using other WM tasks (Schultz & Cole, 2016; Zuo et al., 2018), suggesting generalizability, this investigation should be expanded to examine and compare the potential contribution of poorer functional reconfiguration to cognitive decline across different domains in aging. In addition, the sample size for older adults in this study was only moderate. Future work on a larger sample of older adults, possibly using a set of different cognitive tasks, are needed. With a larger sample of healthy older adults, future work could also subgroup the sample into high and low performers to investigate possible differences in functional reconfiguration associations within subgroups. Furthermore, we only used a cross-sectional design in this study, which may not accurately reflect aging trajectories. Longitudinal studies can be used in future work to investigate changes in functional reconfiguration over time and link them to cognitive decline in aging. We also note that parcellation choice could affect the results from network-based fMRI studies (Zalesky et al., 2010). However, since our results agree with previous studies on functional reconfiguration using different parcellations, these findings are likely to be independent of parcellation choice. This study investigated reconfiguration of static FC at rest and task, but the dynamics of functional network organization are also relevant to task performance (Shine et al., 2016). Further work is required to investigate whether these dynamic reconfigurations are relevant to age-related cognitive decline. Finally, we only considered FC in this study, and future work could link functional reconfiguration with brain activations to further investigate the relevance of this measure to CRUNCH and other theories of cognitive aging such as neural dedifferentiation. Of note, beside functional communication as we and many other researchers assumed, statistical dependence between regions captured by FC could also arise from noncommunicative and/or communicatively detrimental scenarios such as coincidental coactivation (Cole et al., 2019) and shared noise or nonspecific responses. Despite not being easy to reconcile with the positive correlation with behavior in our study, the latter might indeed be a key component in an aging functional organization (Morcom & Henson, 2018) that deserves further elucidation.

## CONCLUSION

We found that global and network-level rest-task functional reconfiguration increased with both age and load, with older adults showing less global reconfiguration between task loads than young adults. Despite these age-related differences, more efficient functional reconfiguration was consistently associated with better task performance in both age groups as well as across different SWM tasks. Furthermore, we found that older adults relied more on internetwork compared to intranetwork reconfiguration for task performance, reflecting reduced functional segregation compared to young adults. Overall, older adults seemed to have less progressive changes in network reconfiguration in response to higher task demands as compared to young adults, possibly reflecting an impaired ability to adapt to increasing cognitive load. These findings highlight the relevance of functional reconfiguration in aging, and the importance of combining measures from both rest and task to study the effects of aging on cognition.

## ACKNOWLEDGMENTS

We would like to thank all participants for their participation in this study.

## SUPPORTING INFORMATION

Supporting information for this article is available at https://doi.org/10.1162/netn_a_00358. Restrictions by the IRB awarded to this project prohibited sharing of our data in public domains. However, the materials that support the findings of this study are available from the corresponding author upon reasonable written request for collaboration.

## AUTHOR CONTRIBUTIONS

Wan Lin Yue: Conceptualization; Formal analysis; Investigation; Methodology; Validation; Visualization; Writing – original draft; Writing – review & editing. Kwun Kei Ng: Conceptualization; Data curation; Formal analysis; Investigation; Methodology; Writing – review & editing. Siwei Liu: Conceptualization; Methodology; Writing – review & editing. Xing Qian: Data curation; Writing – review & editing. Joanna Su Xian Chong: Methodology; Writing – review & editing. Amelia Jialing Koh: Data curation; Project administration; Writing – review & editing. Marcus Qin Wen Ong: Data curation; Project administration; Writing – review & editing. Simon Kang Seng Ting: Data curation; Writing – review & editing. Adeline Su Lyn Ng: Data curation; Writing – review & editing. Nagaendran Kandiah: Data curation; Funding acquisition; Writing – review & editing. B. T. Thomas Yeo: Supervision; Writing – review & editing. Juan Helen Zhou: Conceptualization; Data curation; Funding acquisition; Supervision; Writing – review & editing.

## FUNDING INFORMATION

Juan Helen Zhou and Nagaendran Kandiah, Biomedical Research Council, Singapore, Award ID: BMRC 04/1/36/19/372. Juan Helen Zhou, National Medical Research Council, Singapore, Award ID: NMRC0088/2015. Juan Helen Zhou, National Medical Research Council, Singapore, Award ID: NMRC/CIRG/0048/2018. Juan Helen Zhou, Yong Loo Lin School of Medicine, National University of Singapore. Kwun Kei Ng, Ministry of Health, Singapore, Award ID: MOH-OFYIRG19may-0012.

## Supplementary Material



## TECHNICAL TERMS

Functional connectivity (FC): The putative functional coupling between brain regions measured as the low-frequency temporal synchrony of their blood oxygen level–dependent (BOLD) signals. Network reconfiguration: The reorganization (topological changes) of brain functional networks to meet changes in cognitive demands (e.g., from rest to task).
Efficient network reconfiguration: Low extent of brain functional network reorganization, i.e., high FC similarity between conditions.
Brain functional network segregation: Brain networks maintaining high FC among brain regions of the same network and low FC with those that are not.
FC similarity: The spatial Pearson correlation between the static FC matrices of two conditions (e.g., rest vs. task).
Rest-task FC similarity: FC similarity between resting state and each load of the spatial working memory task.
SWM load: The purported amount of ‘cognitive demands’ imposed by changing the number of dot locations participants had to remember.
Global and network-level FC similarity: FC similarity derived from all brain region pairs or a subset according to network membership, respectively.
Stepwise FC similarity: FC similarity between conditions with progressively increased cognitive demand.
Intra- and internetwork FC similarity: FC similarity derived from brain region pairs belonging to the same or different networks, respectively.
Higher order brain functional networks: Networks more involved in abstract processing and computations of internal and external stimulations, thus ‘higher’ in the functional hierarchy.
Lower order brain functional networks: Networks involved prominently in, but not limited to, sensoriperceptual processing, thus at the ‘lower’ initiation stages along the functional hierarchy.
Efficient network updating: Low extent of brain functional network reorganization, i.e., high FC similarity between conditions.
Compensation by additional recruitment: Putative mechanism to support cognition with a revised circuit with additional neural correlates as the typical one no longer suffices.
