# Supplementary material for: Differential spatial working memory–related functional network reconfiguration in young and older adults
Source: Netw Neurosci. 2024 Jul 1;8(2):395–417. doi: 10.1162/netn_a_00358 (PMC11142455; doi:10.1162/netn_a_00358)
Supplement: Supplementary file 1 [file netn-8-2-395-s001.pdf]

## Table of Contents

*Supplementary Methods* ........ 2

*Supplementary Results* ........ 3

*Supplementary Figure 1. Task paradigm and performance for spatial working memory (SWM) task with distraction and interference.* ........ 5

*Supplementary Figure 2. More efficient global and network-level functional reconfiguration is associated with better performance across spatial working memory (SWM) tasks.* ........ 6

*Supplementary Table 1. Post-hoc statistics on task performance measures in SWM-load task.* ........ 8

*Supplementary Table 2. Post-hoc statistics on global stepwise FC similarity.* ........ 9

*Supplementary Table 3. Statistics for age and load effects on global and network-level functional reconfiguration.* ........ 10

*Supplementary Table 4 (intra-network summary). Post-hoc statistics for age x load interactive effects on network-level stepwise functional reconfiguration. Only statistically significant differences after multiple comparisons adjustment are presented.* ........ 13

Supplementary Table 4 (continued, inter-network summary). ........ 15

*Supplementary Table 5. Association of rest-task FC similarity for each load comparison with SWM-load task accuracy in young and older adults.* ........ 17

*Supplementary Table 6. Association of stepwise FC similarity for each load comparison with SWM-load task accuracy in young and older adults.* ........ 20

*Supplementary Table 7. Association of rest and task FC for each load with SWM-load task accuracy in young and older adults.* ........ 22

*Supplementary Table 8. Association of rest-task FC similarity for each load comparison with SWM-load task accuracy in young adults motion-matched to older adults.* ........ 25

*Supplementary Table 9. Association of stepwise FC similarity for each load comparisons with SWM-load task accuracy in young adults motion-matched to older adults.* ........ 28

*Supplementary Table 10. Association of rest-task FC similarity for each load comparison with SWM-load task accuracy in young and older adults controlling for response time.* ........ 30

*Supplementary Table 11. Association of stepwise FC similarity for each load comparison with SWM-load task accuracy in young and older adults controlling for response time.* ........ 33

*Supplementary Table 12. Association of rest-task FC similarity with SWM-I and SWM-D task response time in young adults.* ........ 35

**Supplementary Methods**

To investigate the generalizability of the behavioral association for functional reconfiguration across different WM tasks, we studied an additional group of young adult participants (N = 32) who performed two other SWM tasks with distraction and interference conditions (SWM-D and SWM-I). These participants were also recruited from National University of Singapore and the community, and the study was approved by the National University of Singapore Institutional Review Board. Written informed consent was obtained from all participants. All participants included in the study had performance above chance level (accuracy > 0.5) for the SWM tasks, as well as fMRI data for resting state and SWM task that met quality control criteria as stated in the main text.

The SWM-D and SWM-I tasks (Supp. Fig. 1) were similar to the SWM-load task, except that no task load modulation was involved, and six dots were always shown simultaneously during encoding. In addition, a face from the CAS-PEAL database (Gao et al., 2007) (gray-scale, Chinese, with neutral expression; image intensity adjusted to be comparable across images) was presented in the middle of the maintenance period. Participants were required to either ignore the face or respond only when it corresponded to the target face (a man in his early 50s) in the SWM-D or SWM-I tasks respectively. Each task trial started with a blank (1s), followed by fixation (0.5s), encoding (0.8s), maintenance (7.2s), face presentation (0.8s), maintenance (7.2s) and retrieval (0.8s). A blank screen was presented between trials with randomly jittered intervals (4s to 8s). The SWM-D and SWM-I tasks comprised two task runs of 18 trials each. Both runs of each task were grouped together, with the order of task type randomized across participants. Individual accuracy (proportion of correct trials out of total trials) and average response time (for correct trials) for each task type were used as task performance measures for all SWM tasks across both age groups.

Resting state and SWM task fMRI data were collected for each participant using a 3T Siemens Prisma Fit MRI scanner (Siemens, Erlangen, Germany) at Centre for Translation MR Research, National University of Singapore. The resting state required participants to fixate on a cross presented on the

center of the screen. Functional scans and T1 structural MRI scans were collected using the same imaging parameters as in the main text. Image preprocessing steps were also the same as in the main text. To obtain FC for SWM-D and SWM-I, task runs for each SWM task type were concatenated. For derivation of FC similarity, face presentation was included as an additional regressor for SWM-D and SWM-I when removing task activations. Since there was only one task load each for SWM-D and SWM-I tasks, FC similarity was computed by taking the correlation between rest and task FC matrices. Associations between FC similarity and task performance were then performed using partial Spearman's correlation, controlling for age, gender, and mean framewise displacement (FD) for rest and task.

**Supplementary Results**

Global FC similarity between rest and task showed negative associations with response time for both SWM-D and SWM-I (Supp. Fig. 2A). However, accuracy in SWM-D and SWM-I was not related to global FC similarity (SWM-D: Spearman's $\rho$ = -0.14, $p$ = 0.453; SWM-I: Spearman's $\rho$ = -0.02, $p$ = 0.908). Putting these together with the associations from the SWM-load task in the main text, the direction of these associations remained consistent across SWM tasks such that better task performance (higher accuracy or faster response times) was correlated with higher FC similarity.

We then studied the contribution of network-level reconfiguration efficiency to the whole brain-level associations observed (Supp. Fig. B-C). Overall, the intra- and inter-network FC similarity of executive control and dorsal attention networks (ECN, DAN) was consistently associated with better task performance across the SWM-D and SWM-I tasks, as well as the SWM-load task as reported in the main text. However, we note that most of these associations did not survive multiple comparisons correction for the 9 networks used ($\alpha = 0.05/9 \simeq 0.006$) for SWM-D and SWM-I (Supp. Table 10), possible due to the smaller sample size (N = 32).

Interestingly, we found that different task performance measures were relevant to functional reconfiguration in the different SWM task types – only response time was associated with

reconfiguration efficiency in SWM-D and SWM-I, while only accuracy was associated with reconfiguration efficiency in SWM-load. We speculate that the differences in task paradigms, in particular the difference in type of presentation (simultaneous versus sequential) and maintenance periods (14s versus 3s), could have contributed to the relevance of different task performance measures to functional reconfiguration in SWM-D and SWM-I tasks compared to the SWM-load task.

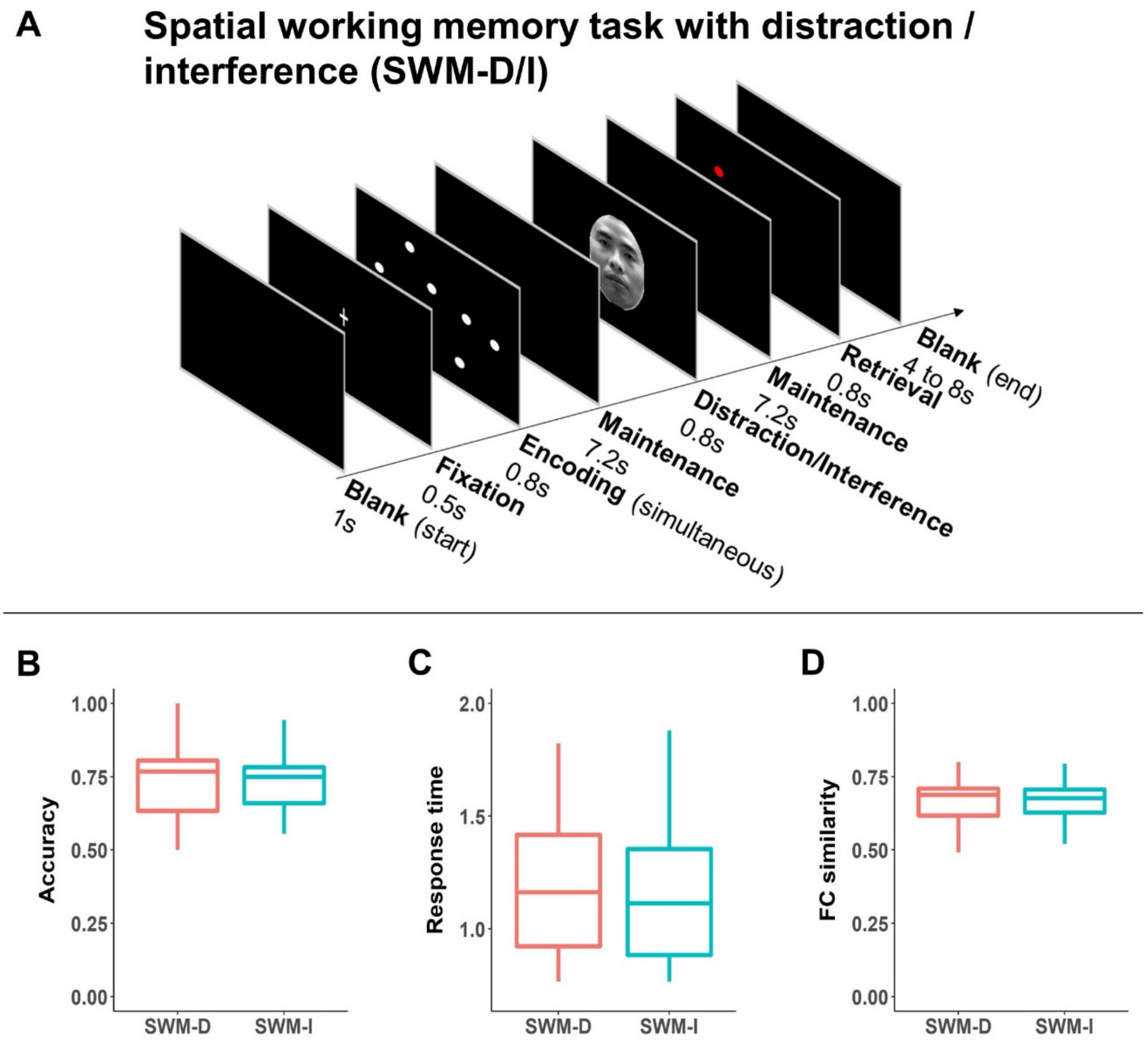


**Supplementary Figure 1. Task paradigm and performance for spatial working memory (SWM) task with distraction and interference. A.** Structure of a single trial in the SWM-distraction (SWM-D) and SWM-interference (SWM-I) tasks. In SWM-D the face presented was viewed passively while in SWM-I, participants were required to respond if a target face was presented. **B-D.** Accuracy **(B)**, response time **(C)** and global functional connectivity (FC) similarity **(D)** in SWM-D and SWM-I tasks.

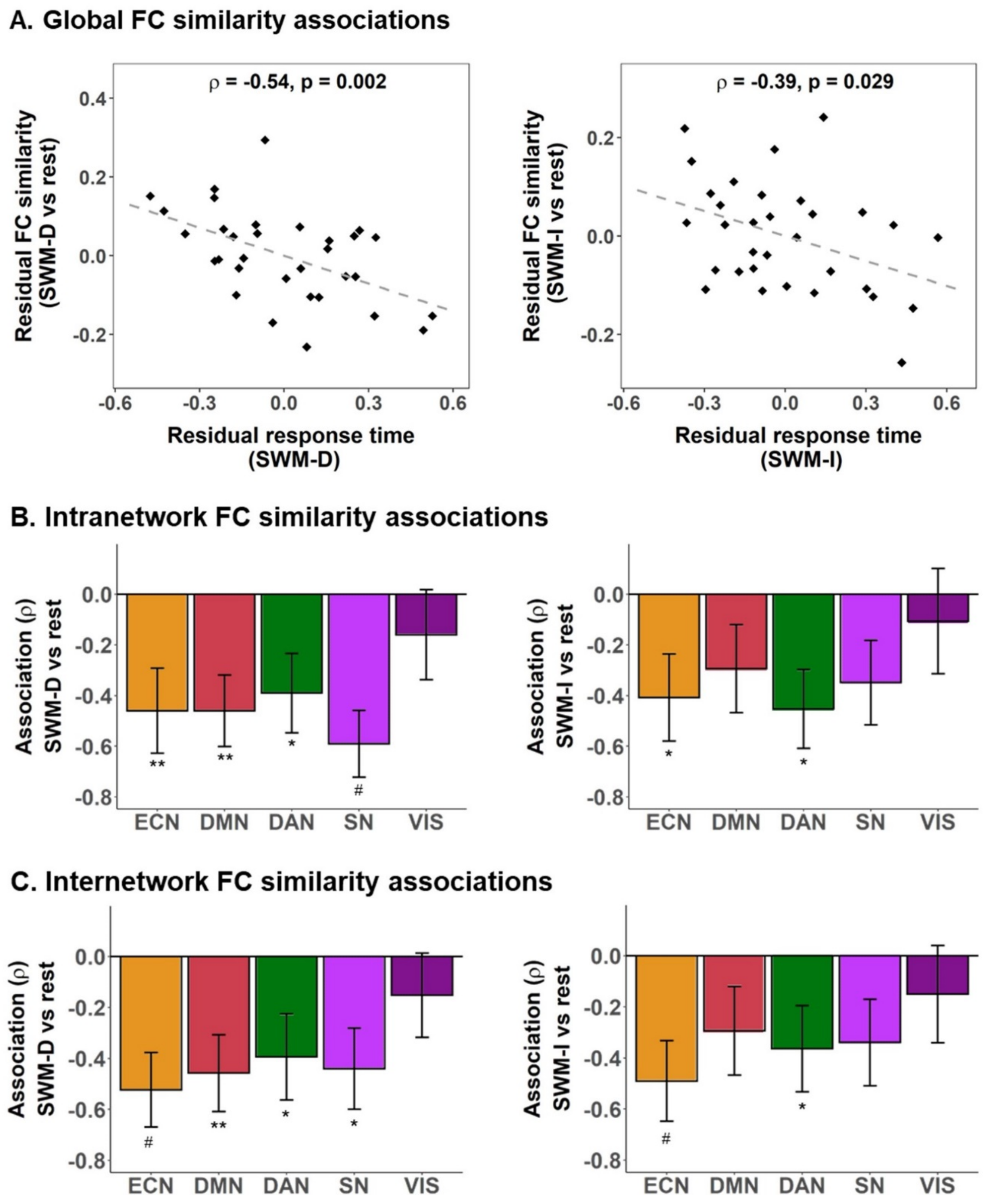


**Supplementary Figure 2. More efficient global and network-level functional reconfiguration is associated with better performance across spatial working memory (SWM) tasks. A.** Higher whole brain FC similarity correlates with shorter response time in the SWM-distraction (SWM-D; left) and SWM-interference (SWM-I; right) tasks. Each dot in the scatter plots represents one participant. **B-C.** Shorter response time in SWM-D (left) and SWM-I (right) tasks was linked to higher intra-network **(B)** and inter-network **(C)** functional connectivity (FC) similarity for higher order and task-relevant

networks. Bars in represent Spearman's correlation between intra-network FC similarity and SWM response time, controlled for age, gender, mean FD (and scanner difference for older adults). Only results from representative networks are illustrated (ECN: executive control network, DMN: default mode network, DAN: dorsal attention network, SN: salience network, VIS: visual network); see Supp. Table 7 for all correlations. Error bars indicate standard error estimated by bootstrap analyses. Asterisks indicate significant correlations ($^{*}$: $p < 0.05$, $^{**}$: $p < 0.01$). Hashes ($^{\#}$) indicate correlations that survived Bonferroni correction for multiple comparisons ($\alpha = 0.05/9 \simeq 0.006$).

**Supplementary Table 1. Post-hoc statistics on task performance measures in SWM-load task.**

| Measure | Age group | Load | Estimate | *p* value |
|---|---|---|---|---|
| Accuracy | Young adults | dot1 vs dot3 | 0.07 | <0.001* |
| | | dot1 vs dot5 | 0.15 | <0.001* |
| | | dot3 vs dot5 | 0.08 | <0.001* |
| | Older adults | dot1 vs dot3 | 0.14 | <0.001* |
| | | dot1 vs dot5 | 0.23 | <0.001* |
| | | dot3 vs dot5 | 0.09 | <0.001* |
| | Older vs young | dot1 | 0.00 | 1.000 |
| | | dot3 | -0.08 | 0.021* |
| | | dot5 | -0.09 | 0.012* |
| Response time | Young adults | dot1 vs dot3 | -0.16 | <0.001* |
| | | dot1 vs dot5 | -0.25 | <0.001* |
| | | dot3 vs dot5 | -0.08 | <0.001* |
| | Older adults | dot1 vs dot3 | -0.25 | <0.001* |
| | | dot1 vs dot5 | -0.27 | <0.001* |
| | | dot3 vs dot5 | -0.02 | 0.952 |
| | Older vs young | dot1 | 0.11 | 0.418 |
| | | dot3 | 0.19 | 0.010* |
| | | dot5 | 0.13 | 0.231 |

Statistics from post-hoc analyses on age group x load interactions from ANOVA using Tukey's honest significant difference (HSD) test. Asterisks (*) indicate significant contrasts.

**Supplementary Table 2. Post-hoc statistics on global stepwise FC similarity.**

| Age group | Load comparison 1 | Load comparison 2 | Estimate | *p* value |
|---|---|---|---|---|
| Young adults | dot1 vs rest | dot1 vs dot3 | -0.0028 | 0.999 |
| | dot1 vs rest | dot3 vs dot5 | -0.0092 | 0.925 |
| | dot1 vs dot3 | dot3 vs dot5 | -0.0063 | 0.990 |
| Older adults | dot1 vs rest | dot1 vs dot3 | -0.15 | <0.001* |
| | dot1 vs rest | dot3 vs dot5 | -0.16 | <0.001* |
| | dot1 vs dot3 | dot3 vs dot5 | -0.0027 | 1.000 |
| Old vs young | dot1 vs rest | | -0.13 | <0.001* |
| | dot1 vs dot3 | | 0.017 | 0.86 |
| | dot3 vs dot5 | | 0.013 | 0.86 |

Statistics from post-hoc analyses on age group x load interactions from ANOVA using Tukey's honest significant difference (HSD) test. Asterisks (*) indicate significant contrasts.

**Supplementary Table 3. Statistics for age and load effects on global and network-level functional reconfiguration.**

| | Network type | Network | Age effects | | Load effects | | Age x Load interactions | |
|---|---|---|---|---|---|---|---|---|
| | | | *F*-statistic | *p* | *F*-statistic | *p* | *F*-statistic | *p* |
| Rest-task FC similarity | Whole brain | | 15.22 | <0.001* | 4.22 | 0.015* | 0.23 | 0.80 |
| | Intra-network | ECN | 27.87 | <0.001# | 2.57 | 0.079 | 0.042 | 0.96 |
| | | DMN | 31.87 | <0.001# | 3.27 | 0.039* | 0.047 | 0.95 |
| | | DAN | 3.82 | 0.053 | 4.72 | 0.0096* | 2.34 | 0.10 |
| | | LN | 0.011 | 0.914 | 0.84 | 0.43 | 0.01 | 0.99 |
| | | SN | 13.76 | <0.001# | 4.54 | 0.012* | 0.30 | 0.74 |
| | | SMN | 0.002 | 0.965 | 1.90 | 0.15 | 0.28 | 0.76 |
| | | TEM | 6.45 | 0.012* | 1.83 | 0.16 | 0.27 | 0.77 |
| | | VIS | 10.97 | 0.0012# | 3.65 | 0.027* | 0.17 | 0.84 |
| | | SUB | 5.54 | 0.020* | 3.66 | 0.027* | 0.76 | 0.47 |
| | Inter-network | ECN | 13.14 | <0.001# | 5.53 | 0.0053# | 0.40 | 0.67 |
| | | DMN | 10.99 | 0.0012# | 5.50 | 0.0045# | 0.23 | 0.80 |
| | | DAN | 6.87 | 0.010* | 3.50 | 0.032* | 0.52 | 0.59 |
| | | LN | 0.065 | 0.80 | 1.04 | 0.36 | 0.23 | 0.79 |
| | | SN | 6.03 | 0.015* | 9.59 | <0.001# | 0.02 | 0.98 |
| | | SMN | 3.02 | 0.085 | 4.96 | 0.075* | 0.38 | 0.69 |
| | | TEM | 2.25 | 0.14 | 1.02 | 0.36 | 0.23 | 0.80 |
| | | VIS | 3.92 | 0.05* | 4.24 | 0.015* | 0.54 | 0.58 |
| | | SUB | 0.002 | 0.97 | 4.75 | 0.0094* | 0.58 | 0.56 |

| | | | | | | | | |
|---|---|---|---|---|---|---|---|---|
| | Whole brain | | 35.84 | <0.001$^{*}$ | 0.93 | 0.394 | 40.37 | <0.001$^{*}$ |
| | | ECN | 16.51 | <0.001$^{\#}$ | 52.91 | | 21.05 | |
| | | DMN | 14.72 | <0.001$^{\#}$ | 43.57 | | 22.64 | |
| | | DAN | 1.83 | 0.18 | 57.60 | | 11.62 | |
| | | LN | 2.41 | 0.12 | 83.21 | | 17.26 | |
| | Intra-network | SN | 1.02 | 0.32 | 35.11 | <0.001$^{\#}$ | 36.33 | <0.001$^{\#}$ |
| | | SMN | 4.93 | 0.028$^{*}$ | 37.61 | | 36.32 | |
| | | TEM | 2.13 | 0.15 | 6.47 | | 8.00 | |
| Stepwise FC similarity | | VIS | 0.36 | 0.55 | 54.21 | | 41.02 | |
| | | SUB | 1.95 | 0.17 | 0.92 | 0.40 | 5.87 | |
| | | ECN | 1.93 | 0.17 | 66.42 | | 36.94 | |
| | | DMN | 0.84 | 0.36 | 67.81 | | 42.23 | |
| | | DAN | 0.44 | 0.51 | 61.92 | | 33.14 | |
| | | LN | 1.36 | 0.25 | 67.50 | | 32.36 | |
| | Inter-network | SN | 0.023 | 0.88 | 54.63 | <0.001$^{\#}$ | 51.65 | <0.001$^{\#}$ |
| | | SMN | 0.63 | 0.43 | 60.54 | | 52.96 | |
| | | TEM | 0.077 | 0.78 | 55.98 | | 46.42 | |
| | | VIS | 0.64 | 0.43 | 72.71 | | 57.70 | |
| | | SUB | 1.13 | 0.29 | 48.04 | | 16.25 | |

Statistics for age group effects, task load effects and age group x load interactions from ANOVA analyses. Asterisks (*) indicate significant associations ($p$ < 0.05) hashes ($^{\#}$) indicate network-level correlations that survived Bonferroni correction for multiple comparisons ($\alpha = 0.05/9 \simeq 0.006$). Whole brain correlation values were not corrected for multiple comparisons. Network abbreviations: executive control network (ECN), default mode network (DMN), dorsal attention network (DAN), limbic network

(LN), salience network (SN), somatomotor network (SMN), temporal-parietal network (TEM), visual network (VIS), subcortical network (SUB).

**Supplementary Table 4 (intra-network summary). Post-hoc statistics for age x load interactive effects on network-level stepwise functional reconfiguration. Only statistically significant differences after multiple comparisons adjustment are presented.**

| Network type | Network | Load A | Load B | Load effect within age group[#] | | | | Age effect within load | | |
|---|---|---|---|---|---|---|---|---|---|---|
| | | | | Young | | Older | | | Older - young | |
| | | | | Estimate | *p* | Estimate | *p* | | Estimate | *p* |
| Intra-network | ECN | D1-Rest | D3-D1 | -.0.025 | 0.012 | -0.13 | <0.001 | D1-Rest | -0.15 | <0.001 |
| | | | D5-D3 | -.0.030 | 0.0026 | -0.13 | <0.001 | D3-D1 | - | - |
| | | | | | | | | D5-D3 | - | - |
| | DMN | D1-Rest | D3-D1 | - | - | -0.14 | <0.001 | D1-Rest | -0.15 | <0.001 |
| | | | D5-D3 | -0.024 | 0.041 | -0.13 | <0.001 | D3-D1 | - | - |
| | | | | | | | | D5-D3 | - | - |
| | DAN | D1-Rest | D3-D1 | -0.037 | <.001 | -0.11 | <0.001 | D1-Rest | -0.70 | 0.001 |
| | | | D5-D3 | -0.050 | <.001 | -0.11 | <0.001 | D3-D1 | - | - |
| | | | | | | | | D5-D3 | - | - |
| | LN | D1-Rest | D3-D1 | -0.096 | <.001 | -0.23 | <0.001 | D1-Rest | - | - |
| | | | D5-D3 | -0.10 | <.001 | -0.23 | <0.001 | D3-D1 | 0.087[&] | 0.030 |
| | | | | | | | | D5-D3 | 0.081[&] | 0.033 |
| | SN | D1-Rest | D3-D1 | - | - | -0.15 | <0.001 | D1-Rest | -0.13 | <0.001 |
| | | | D5-D3 | - | - | -0.15 | <0.001 | D3-D1 | - | - |
| | | | | | | | | D5-D3 | - | - |
| | SMN | D1-Rest | D3-D1 | - | - | -0.12 | <0.001 | D1-Rest | -0.046 | 0.048 |
| | | | D5-D3 | - | - | -0.13 | <0.001 | D3-D1 | 0.071[&] | 0.004 |
| | | | | | | | | D5-D3 | 0.085[&] | <0.001 |
| | TEM | D1-Rest | D3-D1 | - | - | -0.070 | 0.005 | D1-Rest | -0.088 | 0.0026 |

| | | | | | | | | | |
|---|---|---|---|---|---|---|---|---|---|
| | | D5-D3 | - | - | -0.080 | 0.001 | D3-D1 | - | - |
| | | | | | | | D5-D3 | - | - |
| | D1-Rest | D3-D1 | - | - | -0.14 | <0.001 | D1-Rest | -0.10 | <0.001 |
| VIS | | D5-D3 | - | - | -0.15 | <0.001 | D3-D1 | - | - |
| | | | | | | | D5-D3 | - | - |
| | D1-Rest | D3-D1 | - | - | - | - | D1-Rest | -0.065 | 0.015 |
| SUB | | D5-D3 | - | - | - | - | D3-D1 | - | - |
| | | | | | | | D5-D3 | - | - |

Statistics from post-hoc analyses on age group x load interactions from ANOVA using Tukey's honest significant difference (HSD) test. [#]None of the difference between dot3 vs. dot1 and dot 5 vs. dot3 was statistically significant. [&]Older adults had higher FC similarity compared to young adults. Network abbreviations: executive control network (ECN), default mode network (DMN), dorsal attention network (DAN), limbic network (LN), salience network (SN), somatomotor network (SMN), temporal-parietal network (TEM), visual network (VIS), subcortical network (SUB). Condition abbreviations: D1-Rest = dot 1 vs. rest, D3-D1 = dot3 vs. dot1, D5-D3 = dot 5 vs. dot3.

**Supplementary Table 4 (continued, inter-network summary).**

| Network type | Network | Load A | Load B | Young Estimate | Young *p* | Older Estimate | Older *p* | Load | Older - young Estimate | Older - young *p* |
|---|---|---|---|---|---|---|---|---|---|---|
| | | | | **Load effect within age group#** | | | | **Age effect within load** | | |
| Inter-network | ECN | D1-Rest | D3-D1 | - | - | -0.17 | <0.001 | D1-Rest | -0.13 | <0.001 |
| | | | D5-D3 | -0.032 | 0.0024 | -0.17 | <0.001 | D3-D1 | - | - |
| | | | | | | | | D5-D3 | - | - |
| | DMN | D1-Rest | D3-D1 | - | - | -0.17 | <0.001 | D1-Rest | -0.13 | <0.001 |
| | | | D5-D3 | -0.027 | 0.017 | -0.18 | <0.001 | D3-D1 | - | - |
| | | | | | | | | D5-D3 | - | - |
| | DAN | D1-Rest | D3-D1 | - | - | -0.15 | <0.001 | D1-Rest | -0.10 | 0.001 |
| | | | D5-D3 | -0.036 | <0.001 | -0.15 | <0.001 | D3-D1 | - | - |
| | | | | | | | | D5-D3 | - | - |
| | LN | D1-Rest | D3-D1 | -0.030 | 0.0056 | -0.18 | <0.001 | D1-Rest | - | - |
| | | | D5-D3 | -0.041 | <0.001 | -0.18 | <0.001 | D3-D1 | 0.12& | 0.030 |
| | | | | | | | | D5-D3 | 0.11& | 0.033 |
| | SN | D1-Rest | D3-D1 | - | - | -0.18 | <0.001 | D1-Rest | 0.12 | <0.001 |
| | | | D5-D3 | - | - | -0.18 | <0.001 | D3-D1 | - | - |
| | | | | | | | | D5-D3 | - | - |
| | SMN | D1-Rest | D3-D1 | - | - | -0.19 | <0.001 | D1-Rest | -0.11 | <0.001 |
| | | | D5-D3 | - | - | -0.19 | <0.001 | D3-D1 | 0.076& | 0.009 |
| | | | | | | | | D5-D3 | 0.075& | 0.009 |
| | TEM | D1-Rest | D3-D1 | - | - | -0.18 | <0.001 | D1-Rest | -0.12 | <0.001 |
| | | | D5-D3 | - | - | -0.20 | <0.001 | D3-D1 | - | - |

| | | | | | | | | | |
|---|---|---|---|---|---|---|---|---|---|
| | | | | | | | D5-D3 | - | - |
| | | D3-D1 | - | - | -0.21 | <0.001 | D1-Rest | -0.12 | <0.001 |
| VIS | D1-Rest | D5-D3 | - | - | -0.21 | <0.001 | D3-D1 | 0.074[&] | 0.0027 |
| | | | | | | | D5-D3 | 0.083[&] | 0.0015 |
| | | D3-D1 | -0.050 | <0.001 | -0.16 | <0.001 | D1-Rest | - | - |
| SUB | D1-Rest | D5-D3 | -0.044 | <0.001 | -0.16 | <0.001 | D3-D1 | 0.064[&] | 0.040 |
| | | | | | | | D5-D3 | 0.069[&] | 0.034 |

Statistics from post-hoc analyses on age group x load interactions from ANOVA using Tukey's honest significant difference (HSD) test. [#]None of the difference between dot3 vs. dot1 and dot 5 vs. dot3 was statistically significant. [&]Older adults had higher FC similarity compared to young adults. Network abbreviations: executive control network (ECN), default mode network (DMN), dorsal attention network (DAN), limbic network (LN), salience network (SN), somatomotor network (SMN), temporal-parietal network (TEM), visual network (VIS), subcortical network (SUB). Condition abbreviations: D1-Rest = dot 1 vs. rest, D3-D1 = dot3 vs. dot1, D5-D3 = dot 5 vs. dot3.

**Supplementary Table 5. Association of rest-task FC similarity for each load comparison with SWM-load task accuracy in young and older adults.**

| Loads | Network type | Network | Young adults | | Older adults | |
|---|---|---|---|---|---|---|
| | | | Spearman's $\rho$ | $p$ value | Spearman's $\rho$ | $p$ value |
| dot1 vs rest | Whole brain | - | 0.46 | <0.001* | 0.24 | 0.177 |
| | Intra-network | ECN | 0.44 | <0.001# | - | - |
| | | DMN | 0.54 | <0.001# | - | - |
| | | DAN | 0.36 | <0.001# | - | - |
| | | LN | 0.18 | 0.065 | - | - |
| | | SN | 0.38 | <0.001# | - | - |
| | | SMN | 0.28 | 0.004# | - | - |
| | | TEM | 0.31 | 0.001# | - | - |
| | | VIS | 0.29 | 0.002# | - | - |
| | | SUB | 0.16 | 0.095 | - | - |
| | Inter-network | ECN | 0.44 | <0.001# | - | - |
| | | DMN | 0.45 | <0.001# | - | - |
| | | DAN | 0.44 | <0.001# | - | - |
| | | LN | 0.32 | 0.001# | - | - |
| | | SN | 0.44 | <0.001# | - | - |
| | | SMN | 0.30 | 0.002# | - | - |
| | | TEM | 0.24 | 0.014* | - | - |
| | | VIS | 0.29 | 0.003# | - | - |
| | | SUB | 0.20 | 0.037* | - | - |
| dot3 vs rest | Whole brain | - | 0.29 | 0.003* | 0.35 | 0.046* |

| | | | | | | |
|---|---|---|---|---|---|---|
| | | ECN | 0.30 | 0.002# | 0.25 | 0.162 |
| | | DMN | 0.39 | <0.001# | 0.26 | 0.140 |
| | | DAN | 0.30 | 0.002# | 0.30 | 0.082 |
| | | LN | -0.01 | 0.959 | 0.30 | 0.088 |
| | Intra-network | SN | 0.22 | 0.024* | 0.39 | 0.024* |
| | | SMN | 0.27 | 0.005# | 0.22 | 0.217 |
| | | TEM | 0.08 | 0.399 | -0.04 | 0.808 |
| | | VIS | 0.10 | 0.288 | 0.42 | 0.014* |
| | | SUB | 0.24 | 0.013* | 0.11 | 0.533 |
| | | ECN | 0.29 | 0.002# | 0.40 | 0.020* |
| | | DMN | 0.26 | 0.007* | 0.38 | 0.025* |
| | | DAN | 0.28 | 0.003# | 0.36 | 0.038* |
| | | LN | 0.23 | 0.014* | 0.38 | 0.028* |
| | Inter-network | SN | 0.28 | 0.003# | 0.44 | 0.011* |
| | | SMN | 0.10 | 0.309 | 0.32 | 0.062 |
| | | TEM | 0.23 | 0.018* | 0.28 | 0.104 |
| | | VIS | 0.09 | 0.367 | 0.33 | 0.058 |
| | | SUB | 0.21 | 0.026* | 0.34 | 0.049* |
| **dot5 vs rest** | Whole brain | - | 0.05 | 0.627 | -0.02 | 0.889 |

Spearman's correlation between FC similarity and SWM accuracy, controlling for age, gender and mean FD in rest and task (and scanner difference for older adults). Asterisks (*) indicate significant associations ($p < 0.05$) hashes (#) indicate network-level correlations that survived Bonferroni correction for multiple comparisons ($\alpha = 0.05/9 \simeq 0.006$). Whole brain correlation values were not corrected for multiple comparisons. Network abbreviations: executive control network (ECN), default mode network

(DMN), dorsal attention network (DAN), limbic network (LN), salience network (SN), somatomotor network (SMN), temporal-parietal network (TEM), visual network (VIS), subcortical network (SUB).

**Supplementary Table 6. Association of stepwise FC similarity for each load comparison with SWM-load task accuracy in young and older adults.**

| Loads | Network type | Network | Young adults | | Older adults | |
|---|---|---|---|---|---|---|
| | | | Spearman's *ρ* | *p* value | Spearman's *ρ* | *p* value |
| dot3 vs dot1 | Whole brain | - | 0.34 | <0.001* | 0.08 | 0.647 |
| | Intra-network | ECN | 0.39 | <0.001# | - | - |
| | | DMN | 0.29 | 0.002# | - | - |
| | | DAN | 0.38 | <0.001# | - | - |
| | | LN | 0.23 | 0.017* | - | - |
| | | SN | 0.31 | 0.001# | - | - |
| | | SMN | 0.35 | <0.001# | - | - |
| | | TEM | 0.19 | 0.053 | - | - |
| | | VIS | 0.20 | 0.037* | - | - |
| | | SUB | 0.23 | 0.017* | - | - |
| | Inter-network | ECN | 0.36 | <0.001# | - | - |
| | | DMN | 0.23 | 0.018* | - | - |
| | | DAN | 0.34 | <0.001# | - | - |
| | | LN | 0.19 | 0.044* | - | - |
| | | SN | 0.35 | <0.001# | - | - |
| | | SMN | 0.29 | 0.002# | - | - |
| | | TEM | 0.21 | 0.026* | - | - |
| | | VIS | 0.23 | 0.016* | - | - |
| | | SUB | 0.11 | 0.244 | - | - |
| dot5 vs dot3 | Whole brain | - | 0.11 | 0.266 | -0.07 | 0.693 |

Spearman's correlation between FC similarity and SWM accuracy, controlling for age, gender and mean FD in rest and task (and scanner difference for older adults). Accuracy of the higher load is used for load comparisons between loads. Asterisks (*) indicate significant associations ($p < 0.05$) hashes ($^{\#}$) indicate network-level correlations that survived Bonferroni correction for multiple comparisons ($\alpha = 0.05/9 \simeq 0.006$). Whole brain correlation values were not corrected for multiple comparisons. Network abbreviations: executive control network (ECN), default mode network (DMN), dorsal attention network (DAN), limbic network (LN), salience network (SN), somatomotor network (SMN), temporal-parietal network (TEM), visual network (VIS), subcortical network (SUB). The associations for dot1-rest are included in Supp. Table 4 and not repeated here.

**Supplementary Table 7. Association of rest and task FC for each load with SWM-load task accuracy in young and older adults.**

| Loads | Network type | Network | Young adults | | Older adults | |
|---|---|---|---|---|---|---|
| | | | Rest | Task | Rest | Task |
| dot1 | Whole brain | - | -0.06 (0.556) | 0.01 (0.946) | 0.04 (0.812) | 0.24 (0.168) |
| | Intra-network | ECN | -0.02 (0.850) | 0.05 (0.597) | -0.25 (0.152) | **-0.44 (0.011)** |
| | | DMN | 0.13 (0.187) | 0.16 (0.089) | 0.03 (0.856) | 0.06 (0.753) |
| | | DAN | 0.00 (0.971) | 0.16 (0.095) | 0.24 (0.180) | -0.05 (0.775) |
| | | LN | 0.11 (0.257) | 0.06 (0.547) | 0.32 (0.065) | 0.26 (0.133) |
| | | SN | 0.03 (0.765) | 0.17 (0.070) | -0.06 (0.731) | 0.14 (0.425) |
| | | SMN | -0.04 (0.663) | -0.03 (0.777) | -0.25 (0.153) | -0.23 (0.191) |
| | | TEM | -0.14 (0.147) | -0.04 (0.648) | 0.16 (0.367) | 0.11 (0.531) |
| | | VIS | -0.05 (0.573) | -0.03 (0.773) | 0.12 (0.488) | -0.01 (0.948) |
| | | SUB | 0.04 (0.698) | 0.12 (0.219) | 0.06 (0.736) | 0.11 (0.531) |
| | Inter-network | ECN | -0.13 (0.166) | 0.04 (0.712) | 0.16 (0.355) | **0.37 (0.030)** |
| | | DMN | -0.15 (0.125) | -0.18 (0.055) | 0.19 (0.276) | 0.03 (0.885) |
| | | DAN | -0.07 (0.457) | 0.02 (0.802) | -0.11 (0.546) | 0.19 (0.287) |
| | | LN | -0.05 (0.625) | -0.03 (0.738) | 0.26 (0.142) | -0.09 (0.627) |
| | | SN | 0.04 (0.702) | -0.01 (0.892) | -0.16 (0.362) | 0.15 (0.400) |
| | | SMN | 0.04 (0.712) | 0.04 (0.666) | 0.20 (0.248) | 0.10 (0.559) |
| | | TEM | 0.04 (0.716) | -0.06 (0.554) | 0.03 (0.855) | 0.22 (0.212) |
| | | VIS | 0.09 (0.334) | 0.04 (0.708) | -0.10 (0.572) | 0.27 (0.116) |
| | | SUB | 0.16 (0.102) | -0.05 (0.600) | -0.13 (0.455) | 0.12 (0.509) |
| dot3 | Whole brain | - | -0.08 (0.420) | 0.05 (0.631) | -0.05 (0.777) | 0.01 (0.950) |

| | | | | | | |
|---|---|---|---|---|---|---|
| | Intra-network | ECN | -0.11 (0.254) | 0.13 (0.165) | -0.13 (0.462) | -0.02 (0.890) |
| | | DMN | 0.01 (0.899) | 0.19 (0.052) | 0.01 (0.948) | -0.12 (0.507) |
| | | DAN | 0.03 (0.763) | 0.09 (0.343) | 0.05 (0.792) | -0.10 (0.560) |
| | | LN | 0.16 (0.101) | -0.01 (0.940) | 0.08 (0.651) | 0.18 (0.310) |
| | | SN | 0.11 (0.264) | 0.12 (0.219) | -0.22 (0.201) | 0.13 (0.453) |
| | | SMN | -0.14 (0.153) | 0.04 (0.715) | **-0.37 (0.032)** | -0.28 (0.105) |
| | | TEM | -0.10 (0.321) | 0.04 (0.705) | 0.02 (0.912) | 0.13 (0.478) |
| | | VIS | -0.05 (0.604) | 0.15 (0.111) | -0.07 (0.694) | 0.09 (0.607) |
| | | SUB | -0.04 (0.705) | 0.13 (0.184) | 0.05 (0.790) | **0.35 (0.042)** |
| | Inter-network | ECN | 0.01 (0.894) | -0.12 (0.211) | 0.02 (0.924) | 0.21 (0.243) |
| | | DMN | -0.03 (0.738) | -0.11 (0.268) | 0.00 (0.989) | **0.36 (0.040)** |
| | | DAN | -0.06 (0.552) | -0.14 (0.145) | -0.24 (0.173) | -0.01 (0.959) |
| | | LN | -0.09 (0.376) | 0.07 (0.462) | 0.17 (0.335) | 0.17 (0.322) |
| | | SN | -0.06 (0.536) | 0.04 (0.714) | -0.04 (0.812) | 0.04 (0.823) |
| | | SMN | 0.10 (0.323) | 0.00 (0.998) | 0.01 (0.963) | -0.09 (0.609) |
| | | TEM | -0.10 (0.306) | 0.05 (0.604) | -0.15 (0.392) | 0.13 (0.470) |
| | | VIS | 0.14 (0.141) | -0.18 (0.061) | 0.12 (0.511) | 0.05 (0.797) |
| | | SUB | 0.05 (0.608) | 0.11 (0.238) | 0.06 (0.740) | 0.11 (0.552) |
| **dot5** | Whole brain | - | -0.10 (0.296) | -0.13 (0.164) | 0.13 (0.448) | -0.01 (0.977) |
| | Intra-network | ECN | **-0.20 (0.034)** | -0.14 (0.143) | -0.16 (0.373) | **-0.43 (0.012)** |
| | | DMN | -0.04 (0.673) | -0.05 (0.631) | -0.11 (0.549) | -0.20 (0.253) |
| | | DAN | -0.03 (0.766) | -0.12 (0.232) | -0.17 (0.325) | **-0.41 (0.015)** |
| | | LN | -0.03 (0.726) | -0.10 (0.311) | 0.20 (0.259) | 0.04 (0.801) |
| | | SN | **-0.25 (0.010)** | 0.01 (0.916) | -0.28 (0.103) | -0.03 (0.874) |
| | | SMN | -0.13 (0.171) | -0.08 (0.386) | 0.10 (0.585) | -0.17 (0.322) |

| | | | | | | |
|---|---|---|---|---|---|---|
| | TEM | **-0.23 (0.014)** | 0.01 (0.903) | -0.31 (0.078) | 0.22 (0.208) |
| | VIS | -0.19 (0.052) | -0.02 (0.875) | -0.06 (0.746) | -0.15 (0.386) |
| | SUB | -0.13 (0.164) | -0.10 (0.304) | -0.23 (0.200) | -0.02 (0.918) |
| Inter-network | ECN | 0.11 (0.254) | 0.07 (0.500) | -0.16 (0.352) | -0.09 (0.629) |
| | DMN | 0.01 (0.939) | 0.02 (0.831) | -0.06 (0.742) | 0.28 (0.105) |
| | DAN | -0.03 (0.755) | -0.01 (0.949) | 0.06 (0.746) | -0.29 (0.097) |
| | LN | 0.04 (0.705) | 0.07 (0.447) | 0.12 (0.503) | **0.44 (0.011)** |
| | SN | 0.01 (0.954) | -0.14 (0.150) | **0.38 (0.029)** | 0.00 (0.996) |
| | SMN | 0.07 (0.484) | -0.04 (0.703) | -0.20 (0.265) | -0.14 (0.430) |
| | TEM | -0.05 (0.641) | -0.03 (0.742) | 0.12 (0.482) | 0.24 (0.167) |
| | VIS | 0.10 (0.282) | -0.16 (0.096) | 0.33 (0.059) | 0.25 (0.162) |
| | SUB | 0.07 (0.485) | 0.04 (0.684) | 0.20 (0.255) | **0.35 (0.041)** |

Spearman's correlation between rest / task FC and SWM accuracy, controlling for age, gender and mean FD (and scanner difference for older adults). Correlation values are displayed in each column with corresponding p values in brackets. Values in bold are significant at uncorrected $p < 0.05$.

**Supplementary Table 8. Association of rest-task FC similarity for each load comparison with SWM-load task accuracy in young adults motion-matched to older adults.**

| Loads | Network type | Network | Spearman's *ρ* | *p* value |
|---|---|---|---|---|
| dot1 vs rest | Whole brain | - | 0.48 | <0.001[#] |
| | Intra-network | ECN | 0.52 | <0.001[#] |
| | | DMN | 0.56 | <0.001[#] |
| | | DAN | 0.40 | 0.001[#] |
| | | LN | 0.24 | 0.052 |
| | | SN | 0.34 | 0.005[#] |
| | | SMN | 0.25 | 0.047[*] |
| | | TEM | 0.41 | 0.001[#] |
| | | VIS | 0.35 | 0.004[#] |
| | | SUB | 0.10 | 0.430 |
| | Inter-network | ECN | 0.47 | <0.001[#] |
| | | DMN | 0.50 | <0.001[#] |
| | | DAN | 0.48 | <0.001[#] |
| | | LN | 0.46 | <0.001[#] |
| | | SN | 0.45 | <0.001[#] |
| | | SMN | 0.28 | 0.025[*] |
| | | TEM | 0.29 | 0.019[*] |
| | | VIS | 0.29 | 0.019[*] |
| | | SUB | 0.23 | 0.061 |
| dot3 vs rest | Whole brain | - | 0.28 | 0.024[*] |
| | Intra-network | ECN | 0.23 | 0.061 |

| | | | | |
|---|---|---|---|---|
| | | DMN | 0.36 | 0.003# |
| | | DAN | 0.24 | 0.049* |
| | | LN | 0.19 | 0.129 |
| | | SN | 0.16 | 0.205 |
| | | SMN | 0.25 | 0.040* |
| | | TEM | 0.15 | 0.224 |
| | | VIS | 0.24 | 0.057 |
| | | SUB | 0.30 | 0.015* |
| | Inter-network | ECN | 0.32 | 0.010* |
| | | DMN | 0.22 | 0.083 |
| | | DAN | 0.22 | 0.083 |
| | | LN | 0.23 | 0.059 |
| | | SN | 0.22 | 0.080 |
| | | SMN | 0.12 | 0.340 |
| | | TEM | 0.22 | 0.073 |
| | | VIS | 0.12 | 0.327 |
| | | SUB | 0.26 | 0.034* |
| **dot5 vs rest** | Whole brain | - | 0.06 | 0.646 |

Spearman's correlation between FC similarity and SWM accuracy, controlling for age, gender and mean FD in rest and task. Accuracy of the higher load is used for load comparisons between loads. Network-level associations were only investigated if whole brain associations were found to be significant. All significant correlation values ($p < 0.05$) with global signal regression are included. Asterisks (*) indicate significant associations ($p < 0.05$) hashes ($^{\#}$) indicate network-level correlations that survived Bonferroni correction for multiple comparisons ($\alpha = 0.05/9 \simeq 0.006$). Whole brain correlation values were not

corrected for multiple comparisons. Network abbreviations: executive control network (ECN), default mode network (DMN), dorsal attention network (DAN), limbic network (LN), salience network (SN), somatomotor network (SMN), temporal-parietal network (TEM), visual network (VIS), subcortical network (SUB).

**Supplementary Table 9. Association of stepwise FC similarity for each load comparisons with SWM-load task accuracy in young adults motion-matched to older adults.**

| Loads | Network type | Network | Spearman's *ρ* | *p* value |
|---|---|---|---|---|
| dot3 vs dot1 | Whole brain | - | 0.33 | 0.007* |
| | Intra-network | ECN | 0.36 | 0.003# |
| | | DMN | 0.20 | 0.100 |
| | | DAN | 0.42 | 0.001# |
| | | LN | 0.25 | 0.041* |
| | | SN | 0.25 | 0.042* |
| | | SMN | 0.30 | 0.015* |
| | | TEM | 0.21 | 0.094 |
| | | VIS | 0.24 | 0.050 |
| | | SUB | 0.30 | 0.014 |
| | Inter-network | ECN | 0.40 | 0.001# |
| | | DMN | 0.23 | 0.060 |
| | | DAN | 0.32 | 0.009* |
| | | LN | 0.21 | 0.098 |
| | | SN | 0.32 | 0.009* |
| | | SMN | 0.30 | 0.015* |
| | | TEM | 0.29 | 0.020* |
| | | VIS | 0.27 | 0.032 |
| | | SUB | 0.27 | 0.029* |
| dot5 vs dot3 | Whole brain | - | 0.09 | 0.454 |

Spearman's correlation between FC similarity and SWM accuracy, controlling for age, gender and mean FD in rest and task. Accuracy of the higher load is used for load comparisons between loads. Network-level associations were only investigated if whole brain associations were found to be significant. All significant correlation values ($p < 0.05$) with global signal regression are included. Asterisks (*) indicate significant associations ($p < 0.05$) hashes ($^{\#}$) indicate network-level correlations that survived Bonferroni correction for multiple comparisons ($\alpha = 0.05/9 \simeq 0.006$). Whole brain correlation values were not corrected for multiple comparisons. Network abbreviations: executive control network (ECN), default mode network (DMN), dorsal attention network (DAN), limbic network (LN), salience network (SN), somatomotor network (SMN), temporal-parietal network (TEM), visual network (VIS), subcortical network (SUB). The associations for dot1-rest are included in Supp. Table 6 and not repeated here.

**Supplementary Table 10. Association of rest-task FC similarity for each load comparison with SWM-load task accuracy in young and older adults controlling for response time.**

| Loads | Network type | Network | Young adults | | Older adults | |
|---|---|---|---|---|---|---|
| | | | Spearman's *ρ* | *p* value | Spearman's *ρ* | *p* value |
| dot1 vs rest | Whole brain | - | 0.45 | <0.001* | 0.18 | 0.304 |
| | Intra-network | ECN | 0.44 | <0.001# | - | - |
| | | DMN | 0.51 | <0.001# | - | - |
| | | DAN | 0.37 | <0.001# | - | - |
| | | LN | 0.16 | 0.103 | - | - |
| | | SN | 0.38 | <0.001# | - | - |
| | | SMN | 0.25 | 0.009* | - | - |
| | | TEM | 0.30 | 0.001# | - | - |
| | | VIS | 0.30 | 0.002# | - | - |
| | | SUB | 0.18 | 0.068 | - | - |
| | Inter-network | ECN | 0.43 | <0.001# | - | - |
| | | DMN | 0.47 | <0.001# | - | - |
| | | DAN | 0.39 | <0.001# | - | - |
| | | LN | 0.31 | 0.001# | - | - |
| | | SN | 0.45 | <0.001# | - | - |
| | | SMN | 0.26 | 0.006* | - | - |
| | | TEM | 0.28 | 0.003# | - | - |
| | | VIS | 0.30 | 0.001# | - | - |
| | | SUB | 0.20 | 0.041* | - | - |
| dot3 vs rest | Whole brain | - | 0.28 | 0.003* | 0.40 | 0.018* |

| | | | | | | |
|---|---|---|---|---|---|---|
| | Intra-network | ECN | 0.29 | 0.002# | 0.34 | 0.050 |
| | | DMN | 0.36 | <0.001# | 0.31 | 0.077 |
| | | DAN | 0.28 | 0.003# | 0.29 | 0.098 |
| | | LN | -0.02 | 0.855 | 0.30 | 0.084 |
| | | SN | 0.22 | 0.023* | 0.35 | 0.041* |
| | | SMN | 0.27 | 0.005# | 0.28 | 0.113 |
| | | TEM | 0.09 | 0.346 | -0.02 | 0.896 |
| | | VIS | 0.12 | 0.222 | 0.43 | 0.011* |
| | | SUB | 0.26 | 0.006* | 0.10 | 0.560 |
| | Inter-network | ECN | 0.30 | 0.002# | 0.47 | 0.006* |
| | | DMN | 0.24 | 0.014* | 0.35 | 0.043* |
| | | DAN | 0.26 | 0.007* | 0.44 | 0.011* |
| | | LN | 0.22 | 0.023* | 0.40 | 0.021* |
| | | SN | 0.28 | 0.003# | 0.45 | 0.009* |
| | | SMN | 0.11 | 0.249 | 0.32 | 0.065 |
| | | TEM | 0.23 | 0.016* | 0.30 | 0.086 |
| | | VIS | 0.07 | 0.477 | 0.43 | 0.013* |
| | | SUB | 0.22 | 0.021* | 0.36 | 0.035* |
| **dot5 vs rest** | Whole brain | - | 0.04 | 0.662 | 0.00 | 0.982 |

Spearman's correlation between FC similarity and SWM accuracy, controlling for age, gender, response time and mean FD in rest and task (and scanner difference for older adults). Asterisks (*) indicate significant associations ($p < 0.05$) hashes ($^{\#}$) indicate network-level correlations that survived Bonferroni correction for multiple comparisons ($\alpha = 0.05/9 \simeq 0.006$). Whole brain correlation values were not corrected for multiple comparisons. Network abbreviations: executive control network (ECN), default

mode network (DMN), dorsal attention network (DAN), limbic network (LN), salience network (SN), somatomotor network (SMN), temporal-parietal network (TEM), visual network (VIS), subcortical network (SUB).

**Supplementary Table 11. Association of stepwise FC similarity for each load comparison with SWM-load task accuracy in young and older adults controlling for response time.**

| Loads | Network type | Network | Young adults | | Older adults | |
|---|---|---|---|---|---|---|
| | | | Spearman's *ρ* | *p* value | Spearman's *ρ* | *p* value |
| dot3 vs dot1 | Whole brain | - | 0.36 | <0.001* | 0.14 | 0.428 |
| | Intra-network | ECN | 0.41 | <0.001# | - | - |
| | | DMN | 0.29 | 0.002# | - | - |
| | | DAN | 0.38 | <0.001# | - | - |
| | | LN | 0.22 | 0.019* | - | - |
| | | SN | 0.33 | <0.001# | - | - |
| | | SMN | 0.36 | <0.001# | - | - |
| | | TEM | 0.19 | 0.052 | - | - |
| | | VIS | 0.19 | 0.043* | - | - |
| | | SUB | 0.24 | 0.012* | - | - |
| | Inter-network | ECN | 0.38 | <0.001# | - | - |
| | | DMN | 0.24 | 0.014* | - | - |
| | | DAN | 0.35 | <0.001# | - | - |
| | | LN | 0.21 | 0.030* | - | - |
| | | SN | 0.37 | <0.001# | - | - |
| | | SMN | 0.32 | 0.001# | - | - |
| | | TEM | 0.23 | 0.015* | - | - |
| | | VIS | 0.24 | 0.013* | - | - |
| | | SUB | 0.11 | 0.244 | - | - |
| dot5 vs dot3 | Whole brain | - | 0.11 | 0.266 | -0.07 | 0.674 |

Spearman's correlation between FC similarity and SWM accuracy, controlling for age, gender, response time and mean FD in rest and task (and scanner difference for older adults). Accuracy of the higher load is used for load comparisons between loads. Asterisks (*) indicate significant associations ($p < 0.05$) hashes ($^{\#}$) indicate network-level correlations that survived Bonferroni correction for multiple comparisons ($\alpha = 0.05/9 \simeq 0.006$). Whole brain correlation values were not corrected for multiple comparisons. Network abbreviations: executive control network (ECN), default mode network (DMN), dorsal attention network (DAN), limbic network (LN), salience network (SN), somatomotor network (SMN), temporal-parietal network (TEM), visual network (VIS), subcortical network (SUB). The associations for dot1-rest are included in Supp. Table 3 and not repeated here.

**Supplementary Table 12. Association of rest-task FC similarity with SWM-I and SWM-D task response time in young adults.**

| Task type | Network type | Network | Spearman's $\rho$ | $p$ value |
|---|---|---|---|---|
| **SWM-D** | Whole brain | - | -0.54 | 0.002* |
| | Intra-network | ECN | -0.46 | 0.009* |
| | | DMN | -0.46 | 0.008* |
| | | DAN | -0.39 | 0.030* |
| | | LN | -0.32 | 0.072 |
| | | SN | -0.59 | <0.001# |
| | | SMN | -0.22 | 0.217 |
| | | TEM | -0.05 | 0.803 |
| | | VIS | -0.16 | 0.388 |
| | | SUB | -0.37 | 0.038* |
| | Inter-network | ECN | -0.52 | 0.002# |
| | | DMN | -0.46 | 0.009* |
| | | DAN | -0.39 | 0.026* |
| | | LN | -0.36 | 0.044* |
| | | SN | -0.44 | 0.012* |
| | | SMN | -0.30 | 0.095 |
| | | TEM | -0.33 | 0.068 |
| | | VIS | -0.15 | 0.404 |
| | | SUB | -0.33 | 0.069 |
| **SWM-I** | Whole brain | - | -0.39 | 0.029* |
| | Intra-network | ECN | -0.41 | 0.021* |

| | | | |
|---|---|---|---|
| | DMN | -0.29 | 0.102 |
| | DAN | -0.45 | 0.010* |
| | LN | -0.08 | 0.682 |
| | SN | -0.35 | 0.051 |
| | SMN | -0.12 | 0.504 |
| | TEM | 0.30 | 0.095 |
| | VIS | -0.11 | 0.560 |
| | SUB | -0.26 | 0.143 |
| Inter-network | ECN | -0.49 | 0.005# |
| | DMN | -0.29 | 0.103 |
| | DAN | -0.36 | 0.041* |
| | LN | -0.24 | 0.177 |
| | SN | -0.34 | 0.058 |
| | SMN | -0.20 | 0.272 |
| | TEM | -0.06 | 0.754 |
| | VIS | -0.15 | 0.411 |
| | SUB | -0.16 | 0.387 |

Spearman's correlation between rest-task FC similarity and SWM response time, controlling for age, gender and mean FD in rest and task. Asterisks (*) indicate significant associations ($p < 0.05$) hashes (#) indicate network-level correlations that survived Bonferroni correction for multiple comparisons ($\alpha = 0.05/9 \simeq 0.006$). Whole brain correlation values were not corrected for multiple comparisons.
